# Glutathione-Dependent Pathways in Cancer Cells

**DOI:** 10.3390/ijms25158423

**Published:** 2024-08-01

**Authors:** Elena Kalinina

**Affiliations:** T.T. Berezov Department of Biochemistry, Peoples’ Friendship University of Russia (RUDN University), 6 Miklukho-Maklaya Street, 117198 Moscow, Russia; kalinina-ev@rudn.ru

**Keywords:** GSH, glutathione transferase, glutathione peroxidase, S-glutathionylation, cancer cells

## Abstract

The most abundant tripeptide—glutathione (GSH)—and the major GSH-related enzymes—glutathione peroxidases (GPxs) and glutathione S-transferases (GSTs)—are highly significant in the regulation of tumor cell viability, initiation of tumor development, its progression, and drug resistance. The high level of GSH synthesis in different cancer types depends not only on the increasing expression of the key enzymes of the γ-glutamyl cycle but also on the changes in transport velocity of its precursor amino acids. The ability of GPxs to reduce hydroperoxides is used for cellular viability, and each member of the GPx family has a different mechanism of action and site for maintaining redox balance. GSTs not only catalyze the conjugation of GSH to electrophilic substances and the reduction of organic hydroperoxides but also take part in the regulation of cellular signaling pathways. By catalyzing the S-glutathionylation of key target proteins, GSTs are involved in the regulation of major cellular processes, including metabolism (e.g., glycolysis and the PPP), signal transduction, transcription regulation, and the development of resistance to anticancer drugs. In this review, recent findings in GSH synthesis, the roles and functions of GPxs, and GST isoforms in cancer development are discussed, along with the search for GST and GPx inhibitors for cancer treatment.

## 1. Introduction

Glutathione (GSH) is a ubiquitous tripeptide (L-γ-glutamyl-L-cysteinyl-glycine) that is present in the cell cytosol of mammalian tissues at 1–15 mM [1]. It is also found in the endoplasmic reticulum, nuclear matrix and peroxisomes. The functions of GSH are related not only to the control and maintenance of redox homeostasis in cells through its involvement in antioxidant protection and thiol–disulfide exchange of peptides and proteins, redox-dependent regulation of cell signaling, and gene expression but also to the detoxification of toxic compounds and synthesis of eicosanoids [1,2,3]. Changes in the levels of GSH have been observed in various human cancers, and elevated GSH levels in cancer cells are associated with tumor progression and increased resistance to chemotherapeutic drugs [4]. To a sufficient extent, the high level of GSH is supported by the reduction of oxidized glutathione (GSSG), which was catalyzed by glutathione reductase (GR) with the use of NADPH, whose production is accelerated because of metabolic reprogramming in cancer cells [5]. The induction of GSH synthesis is also found in many cancers (e.g., breast, ovarian, lung, head, and neck cancers) [6], but its controlling mechanisms are not clear enough.

In cancer cells, the redox balance is disrupted, leading to enhanced reactive oxygen species (ROS) production due to several factors including activation of oncogenes, aerobic glycolysis, and hypoxia [7,8]. Some anticancer agents (e.g., anthracyclines, alkylating agents, and platinum coordination complexes) also act by increasing ROS production. Cancer cells are able to develop an adaptive antioxidant response to increased ROS levels with the formation of cancer drug resistance to defend their survival, and they keep low ROS thresholds to abolish cell death [9,10]. The role of GSH in the acquisition of drug resistance in different cancers (e.g., melanoma, hepatocarcinoma, bone marrow, breast, colon, pancreatic, and lung cancers) and in the neoplastic progression is supported by its involvement in the detoxification and elimination of carcinogens, as well its antioxidant function.

Among the participants of the antioxidant system, GSH plays a central role in countering oxidative stress [7]. GSH is able to directly scavenge ROS (e.g., hydrogen peroxide, superoxide, and peroxynitrite) [11,12,13] and serves as a redox cofactor for such key enzymes with antioxidant functions, including glutathione peroxidases (Gpxs), glutathione S-transferases (GSTs), and glutaredoxins (Grxs) [14]. GPxs and GSTs reduce hydroperoxides to alcohols and effectively defend against increased oxidative stress by supporting cellular redox homeostasis, which limits ROS to a tumor-promoting level [15]. The family of GPxs (Se- and non-Se-dependent peroxidases) uses GSH as a reducing agent in catalyzing the reduction of H_2_O_2_ or organic hydrogen peroxides, which works with superoxide dismutase and catalase to form an enzymatic antioxidant system that reduces ROS and limits their toxicity. However, each member of the GPx family (GPx1–8) has a different mechanism of action and site of action in maintaining redox balance [16]. In addition, aberrations of expression and polymorphisms of different GPx genes cause their dual role in cancer [17,18].

Three GST superfamilies (canonical soluble, mitochondrial, and membrane-associated isoforms) are among the key antioxidant system and phase II detoxification enzymes maintaining cellular homeostasis [19,20]. GSTs play a cytoprotective role primarily by catalyzing the conjugation of reactive electrophiles generated by cytochrome P450 metabolism, using GSH as a cosubstrate, as well as the reduction of hydroperoxides to eliminate toxic products of xenobiotic biotransformation and oxidative stress [20]. In addition, GSTs are known for their functions in cell signaling, post-translational modification, and resistance to chemotherapeutic agents. Overexpression of GSTs, particularly GSTP1-1, is often considered to be a mechanism of cancer drug resistance [21,22]. GSTs are involved in the regulation of cell viability through S-glutathionylation of important target proteins (for example, peroxiredoxin VI (Prx VI) and p53) [23].

Despite the high quality of the collected data on the importance of GSH, GPxs, and GSTs in malignant transformation, peculiarities of GSH synthesis and the functions of GPxs and GSTs in tumor cells have been poorly understood thus far. In this review, recent findings in GSH synthesis, the roles of functions of GPxs, and GST isoforms in cancer development are discussed, along with the search for GST and GPx inhibitors for cancer treatment. 

## 2. GSH Synthesis in Cancer Cells

### 2.1. γ-Glutamyl Cycle and the Key Enzymes of GSH Synthesis

**Figure 1:**
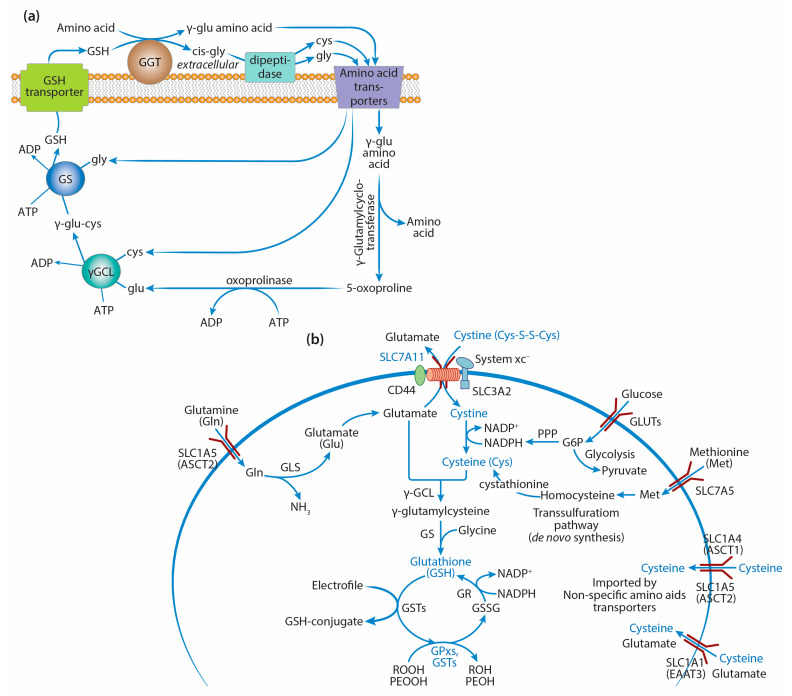
GSH synthesis and transport of precursor amino acids: (**a**) *γ*-Glutamyl cycle of GSH synthesis. γ-Glutamyl transferase (GGT) is localized on the outer side of the cytoplasmic membrane and facilitates the transfer of the γ-glutamyl residue to the neutral amino acid, enabling its transport into the cell. The dipeptide cysteinylglycine, formed as a result of GGT’s action, is cleaved by dipeptidase into cysteine and glycine, which become the substrates for γ-glutamylcysteine ligase (γGCL) and glutathione synthetase (GS). γ-Glutamylcyclotransferase breaks the bond between the γ-glutamyl residue and the amino acid with the formation of a free amino acid and 5-oxoproline, which is decyclized by oxoprolinase with the formation of glutamic acid, the substrate for γGCL. Cysteine and glutamate synthesize γ-glutamylcysteine (GGC) via γGCL, and glycine is added to its C-terminus by GS to form GSH. (**b**) *Transport of GSH precursor amino acids*. Cysteine is a rate-limiting precursor of GSH synthesis and is mainly obtained through cystine uptake via a cystine/glutamate exchange transporter, system xc- (SLC7A11), which is overexpressed in many human cancers. Cysteine also may be generated partially de novo via the transsulfuration pathway or carried by other non-specific amino acid transporters, including SLC1A4 and SLC1A5 (alanine–serine–cysteine transporters, ASCT1/2), and the excitatory amino acid transporter SLC1A1 (EAAT3), which simultaneously acts as a high-affinity, sodium-dependent glutamate carrier. Glutamine enters the cells through several amino acid transporters, including SLC1A5 (ASCT2), which is highly overexpressed in cancer cells. Glutaminase (GSL) produces glutamate from glutamine. GSH, as a cosubstrate of key GSH-related enzymes (GPxs and GSTs), takes part in the detoxification of organic (POOH) or lipid (PEOOH) hydroperoxides into corresponding alcohols and electrophiles into GSH conjugates. *GR* catalyzes the *NADPH*-driven *reduction* of oxidized glutathione (GSSG) to *GSH*.

GSH synthesis involves a two-step ATP-dependent enzymatic reaction (Figure 1a). The first step is catalyzed by γ-glutamylcysteine ligase (γGCL), which ligates cysteine to glutamate to produce γ-glutamylcysteine, which is then combined with glycine by GSH synthetase (GS) to produce GSH [24,25]. The enzyme capable of hydrolyzing the specific bond between glutamic acid and the cysteine residue in the GSH molecule, γ-glutamyl transferase (GGT), is localized on the outer side of the cytoplasmic membrane in particular types of cells and facilitates the transfer of the γ-glutamyl residue to the neutral amino acid, enabling its transport into the cell. The dipeptide cysteinylglycine, formed as a result of GGT’s action, is cleaved by dipeptidase into cysteine and glycine, which become the substrates for γGCL and GSS.

Tumor cells demonstrate considerable changes in gene expression and the activity of enzymes involved in GSH synthesis. The increase in GSH synthesis can be a consequence of metabolic reprogramming of tumor cells associated with redox-dependent signaling, the type of which is largely determined by the GSH/GSSG ratio, allowing GSH to participate in the regulation of cell systems with mutual feedbacks. In particular, it was shown that transduction of the oncogenic signal by phosphatidylinositol 3-kinase (PI3K) could stimulate an increase in the GSH level in breast cancer cells [26]. Under physiological conditions, the activity of γGCL and the content of cysteine are the factors limiting the rate of GSH synthesis [27,28,29,30].

γGCL is a heterodimer consisting of a 73 kDa catalytic subunit (GCLC) and a 31 kDa modulating subunit (GCLM) [31,32], which exerts a regulatory effect on the activity of the catalytic subunit. Mice with a null mutation in the GCLM gene have less than 25% of intracellular GSH. γGCL is inhibited by the product of the synthesis (GSH) via a feedback mechanism [27]. Under the conditions of oxidative stress, the γGCL level in the cell is regulated to a large extent by the transcription factors nuclear erythroid 2-related factor 2 (Nrf2) and nuclear factor kappa-light-chain-enhancer of activated B cells (NF-κB) [24,33].

In various types of cancer, tumor cells demonstrate enhanced expression of the GCLC and GCLM genes. A considerable increase in *GCLC* gene expression was observed in patients with lung cancer [34,35]. The enhanced expression of the *GCLC* gene and high activity of γGCL were shown in patients with squamous-cell carcinoma of the head and neck [36,37]. Examination of patients with colorectal tumors showed an increase in the content of *GCLC* mRNA in 18.8% of adenomas and 84.2% of carcinomas [38]. High activity of γGCL was found in patients with renal cell carcinoma [39]. An increase in GSH content and the level of GCLC gene transcription induced by the proteasome inhibitor lactacystin was shown in HT29 colorectal adenocarcinoma cells. This increase in the level of GCLC protein depended on the p38 MAPK. It was shown that ROS generation in MG63 osteosarcoma cells increased under the effect of prostaglandin (15dPGJ2), causing activation of p38 MAPK and subsequent phosphorylation of Akt kinase [40], the key enzyme of the PI3K/Akt signaling pathway, which participates in the regulation of cells’ proliferation and survival. This pathway is involved in the activation of transcription factors Nrf2 and early growth response protein 1 (Egr1), which leads to the enhanced expression of the *GCLC* gene. Enhanced expression of the *GCLM* gene in tumor cells could be associated with the development of drug resistance [41,42]. In addition, mutations in the *GCLM* gene lead to delayed tumor onset in models of breast sarcoma and lymphoma, making *GCLM* an effective pharmacological target for the control of chemotherapeutic resistance in these malignant neoplasms [43,44,45]. The authors believe that the data allow the *GCLM* gene to be considered a promising target for controlling drug resistance in these types of cancer.

The second key enzyme of GSH synthesis, GS, is a dimer of two identical subunits containing two domains: one is intended for ATP binding, and the other is a catalytic center. The binding of ATP to the enzyme requires two magnesium ions (Mg^2+^) as cofactors. In humans, the *GSS* gene defects are passed on through autosomal recessive inheritance. Mutations in the *GSS* gene can cause the onset of metabolic acidosis of varying severity, 5-oxoprolinuria, exaggerated hemolysis, and impaired function of the central nervous system. Determination of GS activity in such patients has shown its correlation with the level of GSH [43]. GS deficiency can occur in the absence of mutations in the *GSS* gene and be a consequence of impaired splicing [46]. Mutations can be combined, such as mutations in the *GSS* gene and in the *OPLAH* gene encoding 5-oxoprolinase [47].

Using functional screening and transcriptome analyses, GS was found to be a potential regulator of radioresistance through ferroptosis in glioma cells [48]. High GS levels were closely related to poor prognosis and relapse in patients with glioma. The depletion of GS led to the disruption of GSH synthesis, thereby causing the inactivation of glutathione peroxidase 4 and the accumulation of iron, enhancing the induction of ferroptosis upon radiotherapy treatment. A CRISPR/Cas9-based gene technique carried out on orthotopic tumors in mice resulted in high *GSS* gene editing efficiency in glioblastoma (up to 67.2%), with negligible off-target gene editing [48].

The onset and development of malignant neoplasms can be accompanied by changes in GS activity. Enhanced activity of GS was found in patients with recurrent bladder cancer and colorectal cancer [49,50]. However, despite these and other works available in the literature reporting enhanced activity of GS in tumor cells, the role of this enzyme in malignant neoplasms remains poorly understood. The activity of the membrane-bound enzyme GGT supplying γ-glutamyl residues is of great importance for the synthesis of GSH. The heterodimeric GGT glycoprotein, consisting of two subunits, is most often expressed on the luminal surfaces of the secretory cells, such as bile ducts and proximal convoluted tubules [51]. The GGT activity increases considerably under oxidative stress, especially in tumor cells with high levels of metabolism, and correlates with the level of GSH [52]. Moreover, GGT expression is reportedly related to drug resistance, possibly because a wide range of drugs are conjugated with GSH, the availability of which is influenced by GGT activity. GGT has been proposed as a biomarker of carcinogenesis and tumor progression, given that GGT activity is important during both the promotion and invasion phases in cancer cells [53]. While serum GGT activity is commonly used as a quick, inexpensive, and reliable means of assessing liver function, recent epidemiological studies have shown that it may also be an indicator of an increased risk of metastatic prostate cancer and advanced urothelial carcinoma [54,55,56]. GGT inhibition is one of the approaches to decrease the intracellular GSH content in order to increase tumors’ sensitivity to different chemotherapeutic agents. The most frequently used GGT inhibitors are glutamate analogs (e.g., acivicin, L-azazerin, 6-diazo-5-oxo-1-norleucine) and boronate derivatives [52,57]. Unfortunately, competitive inhibitors such as glutamic acid analogs are highly toxic. In addition to these preparations, the use of noncompetitive inhibitors has been proposed, such as OU749, which has low toxicity and considerably increases the therapeutic sensitivity of tumor cells by decreasing GSH and cysteine levels [53]. The search for novel GGT inhibitors among the GSH conjugates, which may include S-geranylgeranyl-L-glutathione and darinaparsin, provides some promise [53,58].

### 2.2. GSH Synthesis and Precursor Amino Acids

The rate of GSH synthesis is largely determined by the concentrations of three amino acids: glutamic acid, cysteine, and glycine, with cysteine being the most essential (Figure 1b). Cysteine is formed mostly through transsulfuration of amino acids. According to this pathway, methionine is activated by ATP via methionine adenosyltransferase and is converted into S-adenosylmethionine (SAM), which is a donor of the methyl group necessary for the methylation of many substrates [59]. SAM hydrolysis is accompanied by the formation of homocysteine, which is converted into cystathionine during catabolism, followed by the production of cysteine and α-ketobutyrate [60]. Enhanced transsulfuration has been shown in tumor cells. The cells of renal cell carcinoma with a low proliferative rate have higher levels of serine, homocysteine, SAM, and S-adenosylhomocysteine [61,62]. Tumor cells with a high degree of proliferation contain increased amounts of α-hydroxybutyrate and products of methionine metabolism, i.e., S-adenosylhomocysteine and homocysteine. It is assumed that the levels of cysteine at the early and late stages of aggressive renal cell carcinoma are preferentially supplemented through transsulfuration [61,62].

Cysteine can also enter tumor cells from the microenvironment via the sodium-independent cystine/glutamate antiporter, which is known as an xc– system or xCT encoded by the *SLC7A11* gene [63]. The heterodimeric cystine/glutamate antiporter consists of the transmembrane protein SLC7A11 linked by a disulfide bond to the regulatory protein SLC3A2. The SLC7A11 protein has 12 transmembrane domains and mainly imports cystine into the cell (in exchange for glutamate), which is reduced to cysteine by cystine reductase [64]. Thus, in addition to synthesis via the transsulfuration pathway, tumor cells can obtain cysteine through this transport pathway, replenishing the intracellular content of Cys necessary for GSH synthesis.

The *SLC7A11* gene required for the proliferation of prostate cancer, breast cancer, renal carcinoma, and colon cancer cells, as has been shown, determines the high content of the light chain (SLC7A11 protein) of system xc– in the membranes of these tumor cells [64]. Tumor stem cells were shown to have higher levels of this transporter, correlating with the GSH content in these cells [65]. The functional activity of the cystine/glutamate antiporter xCT facilitates the oncogenic transformation of RAS proteins and makes noticeable contributions to the maintenance of the intracellular redox balance in tumor cells [66]. Expression of the *SLC7A11* gene may be regulated by some transcriptional factors. Nrf2 and activating transcription factor 4 (ATF4) control its enhanced levels in tumor cells [67,68,69]. ATF4 promotes *SLC7A11* transcription in response to amino acid starvation, endoplasmic reticulum (ER) stress, and hypoxia due to the increase in ATF4 translation [68,70]. ATF4 activates the expression of stress-response genes, including *SLC7A11*, through its binding to the amino acid response element (AARE) [70]. The Kelch-like ECH-associated protein 1 (Keap1)-Nrf2-activator protein-1 (AP-1)/antioxidant response element (ARE) signaling pathway increases the transcription of genes responsible for resistance to oxidative stress, including *SLC7A11* [71,72]. Oxidative stress impairs Nrf2’s degradation by Keap1 and allows Nrf2 to bind to ARE, which is involved in antioxidant defense and redox maintenance. In tumor cells, Keap1 inactivation promotes ferroptosis resistance following activation of the SLC7A11/cysteine/GSH axis by stabilizing Nrf2 and its target genes [68]. *SLC7A11* expression can also be repressed by transcription factors that take part in tumor suppression. For example, p53 directly represses *SLC7A11* transcription and activates ferroptotic cell death through various ferroptosis inducers [73]. Activating transcription factor 3 (ATF3), a common stress sensor, binds to the *SLC7A11* promoter under basal conditions and represses its expression independent of p53. Erastin treatment or cystine deficiency induces *SLC7A11* expression, whereas upregulating ATF3 suppresses its expression, depletes intracellular GSH, and promotes ferroptosis in cancer cells [74,75]. 

Some types of tumor cells (e.g., leukemia, lymphoma) are unable to produce cysteine. Therefore, the amino acid should enter such cells from the microenvironment to sustain their growth. When the content of cysteine in the cells decreases, it is replenished via system xc– due to cystine excretion by the neighboring cells (i.e., fibroblasts activated by macrophages). Therefore, the cystine/glutamate antiporter is considered to be a potential target for treating malignant neoplasms when the growth and survival of tumor cells depend on the external supply of amino acids [76,77].

Cysteine can also be imported into cells from the extracellular milieu by non-specific transporters, such as excitatory amino acid transporter 3 (EAAT3) and alanine–serine–cysteine transporters 1 and 2 (ASCT1/2). These transporters are also associated with transporting other amino acids, e.g., glutamine and glutamate. A limited number of studies have examined these transporters in cancer: Their overexpression has been observed in different cancer cells, and it was associated with increased chemoresistance in colorectal and prostate cancers [78].

In addition to cysteine, GSH synthesis depends on the levels of glutamine and glutamic acid. The availability of glutamine affects GSH synthesis via three different mechanisms: In the first one, glutamine is the main source of glutamic acid formed by glutaminases (GSLs) 1 and 2 [79]. It has been shown that glutaminase activity is strictly regulated to preserve the intracellular levels of glutamate required for GSH synthesis [79]. Glutamine can be transported by several different amino acid transport systems. Among them, there is solute carrier family 1, where protein 5 (SLC1A5/ASCT2) is the most frequently overexpressed transporter protein in the different human tumor cells dependent on extracellular glutamine [80]. The glutamine and GSH levels decrease in cells with low expression of the gene of this transporter, and the activity of glutaminases 1 and 2 also decreases. This system is regulated by the oncogenic transcription factor cMyc in prostate cancer and lymphoma cells [80,81]. It was shown that miR23a (miroRNA23a) and miR 24b could suppress the expression of the *SLC1A5* gene by reducing glutamine metabolism in the tumor [81]. Repression of these microRNAs by the oncogenic factor cMyc led to enhanced *GLS* gene expression and more intensive glutamine catabolism in melanoma cells. The second mechanism of glutamine’s effect on GSH levels is associated with its role in the maintenance of the GSH content by preserving the level of NADPH(H^+^) formation via regulation of the malate content [79]. Malate production from glutamine can occur via two pathways: In the first pathway, malate generated from glutamine is transported from mitochondria to the cytoplasm and converted by the malic enzyme into pyruvate, reducing NADP^+^ to NADPH(H^+^). In the second pathway associated with the malate–aspartate shuttle system, aspartate formed in the mitochondrial matrix during the re-amination of glutamate, and oxaloacetate is transported from mitochondria to the cytosol. In the cytosol, aspartate is transaminated into oxaloacetate under the influence of AST (aspartate aminotransferase) and then transformed into malate, followed by the production of pyruvate and NADPH(H^+^) [82].

The third mechanism of action of glutamine on GSH synthesis is associated with the transport of cystine into the cell. The cystine–glutamate transport system xc– depends on glutamate’s availability in the cells because one glutamate molecule is exchanged for one cystine molecule. It has been proven experimentally that glutamine and glutamate levels are crucially important for the maintenance of the GSH levels necessary for cell viability [79]. In addition to glutamate and cysteine, GSH synthesis depends on the availability of glycine, which is an amino acid required for many metabolic processes, including the synthesis of purines (e.g., adenine, guanine) in rapidly dividing tumor cells. A deficiency of serine and glycine decreases GSH synthesis and increases ROS generation in the tumor cell culture. Glycine is a nonessential amino acid that can be synthesized from serine. In some cases, serine can be obtained via transsulfuration. The tumors wherein this pathway is impaired are characterized by the enhanced accumulation of 3-phosphoglycerate [83]. It was shown experimentally that increased levels of glycine facilitated oncogenesis [84]. In NCI60 human tumor cells, it was shown that glycine consumption and the expression of enzyme genes necessary for glycine biosynthesis were correlated with the rate of cell proliferation.

The high GSH/GSSG ratio in tumor cells can be explained by the higher activity of the pentose phosphate pathway (PPP), leading to NADPH(H^+^) production [85]. Activation of the PPP, enhanced levels of GSH, and intermediate metabolites of the PPP were demonstrated in renal cell carcinoma [86]. A feedback loop of the PPP and the PI3K/Akt signaling pathway, along with the increased GSH levels, drives regorafenib resistance in hepatocellular carcinoma cells, and targeting this feedback loop could be a promising approach to overcome drug resistance [87]. The PPP is the main intracellular pathway leading to the formation of pentoses necessary for the synthesis of nucleic acids, coenzymes (e.g., NAD, FAD), macroergic compounds (e.g., ATP, GTP), and cyclic nucleotides (e.g., cAMP, cGMP). The second biosynthetic feature of the PPP is the production of NADPH(H^+^), making up to 65–70% of its total content in the cell, which is necessary for the biosynthesis of fatty acids, cholesterol, bile acids, and steroid hormones. Growing tumor cells require all of these compounds together with GSH, the pool of which is also replenished by GR catalyzing GSSG reduction via NADPH(H^+^) [24].

The observed increase in GSH levels is due not only to the higher levels of ROS production in most tumor cells but also to the fact that some of the classical tumor promoters also activate GSH synthesis and turnover mechanisms (e.g., Nrf2) [25,88]. In tumors associated with mutated KEAP1, there are increases in Nrf2 activity and the flux of glutamine to glutamate for GSH synthesis [89]. Nrf2 regulates glutathione metabolism not only through the induction of enzymes in glutathione synthesis (e.g., GCLC, GCLM, GS) but also through the expression of enzymes that are responsible for glutathione utilization and redox cycling reduction (e.g., GR, SOD1, catalase, and several GPxs and GSTs) [90].

Recent research revealed that estrogen-related receptors (ERRs) act as novel redox sensors and effectors of an ROS defense program in breast cancer. Specifically, it was demonstrated that ERRs control glutamine utilization and glutathione antioxidant production [91]. In addition, the expression of the *GCLM* and *SLC7A11* genes is regulated by hypoxia-inducible factor 1-alpha (HIF1α), controlling GSH synthesis in hypoxic environments [92].

## 3. Glutathione Peroxidases and Antioxidant Defense in Cancer Cells

### 3.1. Glutathione Peroxidases and Decomposition of *Hydroperoxides*

The family of GPxs reduces hydroperoxides to their corresponding alcohols (water in the case of H_2_O_2_), mainly using GSH as a cosubstrate [93,94]. In mammals, this family comprises eight members (GPx1–8), five of which are selenoproteins in humans (GPx1–4 and Gpx6). The other three isoforms (GPx5, -7, and -8) contain a cysteine (Cys) instead of a selenocysteine (Sec) moiety [16,95].

In the decomposition reaction of H_2_O_2_ or other organic peroxides (ROOH), two molecules of GSH reduce the substrate to H_2_O or the corresponding alcohol (ROH):

2GSH + ROOH → GSSG + ROH + H_2_O

Oxidized glutathione is reduced by GR, completing the cycle:

GSSG + NADPH(H^+^) → 2 GSH + NADP^+^

The reaction mechanism differs between individual GPx isoforms. In general, the reaction mechanism has an oxidation and a reduction part [96]. In the enzyme’s active center, where the Glu, Try, and Asp residues form a highly nucleophilic region, oxidation of the active site selenocysteine (Rr-SeH) or cysteine (R-SH) occurs after binding the peroxide, which leads to the formation of a selenenic acid (Pr-SeOH) derivative in selenium-containing isoforms. The selenenic acid is then reduced back to selenol (Pr-SeH) via a two-step process that begins with a reaction with GSH to form Pr-SeSG, which is reduced by the second GSH molecule, releasing GS-SG [97,98]. The reaction with H_2_O_2_ as a substrate is as follows:

Pr-SeH + H_2_O_2_ → Pr-SeOH + H_2_O

Pr-SeOH + GSH → Pr-SeSG + H_2_O

Pr-SeSG + GSH → Pr-SeH + GSSG

GPx1, -2, -3, -5, and -6 are homotetramers, which could determine their specificity for hydrogen peroxide. GPx4, -7, and -8 are monomers. This structure probably enables their reaction with more complex lipid hydroperoxides, but this has been proven only for GPx4 [96,98]. GPx1 is ubiquitous in the cytosol and mitochondria, GPx2 in the cytosol of epithelium cells, and the extracellular isoform GPx3 in the plasma, reducing H_2_O_2_ and free fatty acid peroxides in the aqueous phase [99]. GPx4 mainly protects membranes by reducing phospholipid and cholesterol peroxides [99,100]. GPx5 (CysGPx isoform) is a secretory enzyme of the epididymis. GPx6 is a human selenoprotein and is formed by the olfactory epithelium. GPx7 and GPx8 are also CysGPx isoforms with low peroxidase activity. Aberrant expression of GPx isoforms in multiple cancers is closely related to oncogenesis and cancer progression [101].

### 3.2. Mammalian Glutathione Peroxidases

#### 3.2.1. GPx1

The *GPX1* gene is located on chromosome 3p21.31, 1178 base pairs in size, and contains two exons; it has five transcript variants [102]. The purified active mammalian GPX1 protein is a homotetramer consisting of four identical subunits with a molecular weight of 22–23 kDa [103]. *GPX1* is ubiquitously expressed in many tissues, mainly distributed in the liver, lungs, and kidneys, and plays a fundamental role in the regulation of intracellular ROS levels by reducing H_2_O_2_, lipid hydroperoxides (PLOOH), and peroxynitrite (ONOO^−^) [104].

GPx1 is closely related to tumorigenesis, mainly due to its role in eliminating hydroperoxides; it is highly expressed in most cancers and has higher expression in tumor tissues, including skin melanoma, testicular germ-cell tumors, glioblastoma multiforme, pancreatic adenocarcinoma, thyroid cancer, renal papillary cell carcinoma, acute myeloid leukemia, endometrial cancer, low-grade glioma, and ovarian serous cystadenocarcinoma [105]. GPx1 may play opposite roles in different types of cancers and can act as a tumor suppressor or promoter (Table 1). In most types of cancer, GPx1 acts as a tumor promoter by regulating the proliferation, invasion, migration, apoptosis, immune response, and drug sensitivity of tumor cells [18]. For example, *GPX1* overexpression prevents ceramide production and partially inhibits apoptosis in doxorubicin-treated human breast carcinoma cells [106]. In esophageal cancer and salivary adenoid cystic carcinoma cells, *GPX1* expression can promote invasion, migration, proliferation, and cisplatin resistance. Since NF-κB transcriptionally activates GPx1, vitamin D can inhibit the NF-κB pathway and *GPX1* expression to reduce tumor malignancy [107,108]. However, several studies have demonstrated the role of GPx1 as a tumor suppressor in some pancreatic and gastric cancers [109,110].

GPx1 is downregulated in most pancreatic cancer cell lines. *GPX1* silencing drives a mesenchymal transition phenotype and gemcitabine resistance by activating the ROS-mediated Akt/GSK3β/Snail signaling axis [18]. Pancreatic ductal adenocarcinoma cells can induce protective autophagy via the activation of ROS/AMP-activated protein kinase (AMPK) signaling and GPx1 degradation to survive in a glucose-starved tumor microenvironment [117].

#### 3.2.2. GPx2

GPx2 is a homotetramer the same size as GPx1, located in the cytosol. In human tissues, *GPX2* mRNA is detected in the liver, stomach, small intestine, and colon and is not detected in the uterus, placenta, or lungs. The *GPX2* gene is located on chromosome 14 and is expressed predominantly in the gastrointestinal tract [118]. Similar to GPx1, GPx2 reduces oxidative DNA damage by reducing hydroperoxides, and together with GPx1, Prxs, and catalase, it is included in the system of major hydroperoxidases. GPx2 is upregulated in most tumor cells [101]. It is conceivable that high *GPX2* expression reflects tumor malignancy, and it is a significant factor in poor prognosis for cancer patients. For example, the estimation of *GPX2* expression in specimens acquired from 351 patients with lung adenocarcinoma who underwent surgery at Kyushu University from 2003 to 2012 revealed that its high expression (*n* = 175, 49.9%) was significantly correlated with male sex, smoking, advanced pathological stage, and the presence of pleural, lymphatic, and vascular invasion [119]. Patients with high *GPX2* expression exhibited significantly shorter recurrence-free survival and overall survival. It has also been shown that *GPX2* overexpression contributes to the initiation, development, and spread of lung, hepatocellular, colon, and prostate cancers [120,121,122,123]. It was found that overexpression of *GPX2* promotes proliferation and invasion, prevents the apoptosis of LNCaP and 22RV1 cells, and triggers the activation of the Wnt/β-catenin and epithelial–mesenchymal transition (EMT) pathways, which are related to the occurrence and development of prostate cancer [123]. In contrast, it has been found that the loss of GPx2 in breast, bladder, and esophageal carcinomas led to tumor progression and worse prognosis [124]. Moreover, GPx2 knockout in mice resulted in intestinal tumorigenesis and sensitized their skin to cancer by irradiation [125]. It has been suggested that *GPX2* expression is tumor-stage-dependent, and upregulation of GPx2 in early-stage carcinomas might protect tumor cells from the effects of ROS on oncogenic signaling, leading to neoplastic progression. The loss of GPx2 in breast cancer cells was assumed to increase ROS levels, thereby activating HIF1α-dependent signaling, which causes vascular malfunction, resulting in hypoxia and metabolic heterogeneity. HIF1α suppresses oxidative phosphorylation and stimulates glycolysis (the Warburg effect) in the tumor [124].

#### 3.2.3. GPx3

The highly conserved selenoprotein and extracellular glycoprotein GPx3, secreted by the basolateral membrane of renal proximal tubule cells, is predominantly present in the extracellular fluid and has specificities similar to those of GPx1, catalyzing the reduction of H_2_O_2_ and organic hydroperoxides [125]. Unlike GPx1, GPx3 can also utilize soluble lipid hydroperoxides as substrates, similar to GPx4. The human *GPX3* gene consists of five exons in the 5q32 region of chromosome 5 and encodes a 23 kDa protein that forms a homotetramer [102].

Like GPx1, GPx3 exhibits a dual role in cancer, and these seemingly contradictory results may be closely related to ROS [126]; it serves as a pro-survival protein in myeloid leukemia and as a tumor suppressor in lung, ovarian, and gastric cancers [127,128,129,130]. In early-stage cancer and precancerous lesions, decreased expression of *GPX3* and increased ROS production promote cancer development. In melanoma, the upregulation of GPx3 plays a role in regulating ROS levels by inhibiting the expression of HIF1α [131,132], which is upregulated in various human cancers and plays a key role in driving tumor growth, invasion, and metastasis. *GPX3* expression is significantly reduced in various tumor tissues, including breast cancer, colon adenocarcinoma, head–neck squamous-cell carcinoma, kidney renal clear-cell carcinoma, lung adenocarcinoma, and stomach adenocarcinoma, and it has good diagnostic accuracy (AUC > 0.75, up to 0.9), which is associated with higher stages and lymph node metastasis, as well as poorer prognosis. The reduced expression of *GPX3* may be the result of epigenetic inheritance mechanisms such as DNA methylation and histone modification [133]. Some results suggest that GPx3 plays a complicated role in the tumor microenvironment, simultaneously promoting metastasis and chemotherapy resistance in human cancers due to enhancing the removal of H_2_O_2_ and lipid hydroperoxides from the extracellular tumor environment [133]. Polymorphisms of the gene encoding *GPX3* are often responsible for the downregulation of gene transcription, resulting in markedly decreased plasma activity of GPX3, and are positively associated with cancer development. Regarding *GPX3* rs8177412 polymorphism, the gene variant that confers lower expression is associated with a significant increase in the risk of upper urothelial carcinoma. Therefore, patients with Balkan endemic nephropathy (BEN) carrying a variant *GPX3* genotype should be more frequently monitored for the possible development of upper-tract urothelial carcinoma [134]. The presence of the *GPX3* rs736775 C allele is linked to the survival outcomes of patients with colorectal cancer [135]. The expression of *GPX3* rs736775 in patients with gastric cancer undergoing adjuvant chemotherapy with platinum and fluorouracil has been associated with enhanced overall survival. It has been suggested that *GPX3* rs736775 be regarded as a potential prognostic marker [136]. In the Taiwanese population, the expression of *GPX3* rs3805435 and rs3828599 showed a significant association with the risk of developing gastric cancer [137]. 

Modifications in Gpx3 play a role in the regulation of various signaling pathways in cancer. In stomach adenocarcinoma cells, GPx3 inhibited the level of pyrimidine metabolism via the ROS/AMPK/mTOR signaling pathway, which could affect the migration and invasive ability of these cancer cells and provide ways to reduce their drug resistance [138]. In lung cancer cells, GPx3 acts as an inhibitor of the proliferation, migration, and invasion of tumor cells by suppressing ROS-mediated NF-κB signaling [139]. In pancreatic adenocarcinoma, GPx3 represses cell proliferation, which regulates the JNK/c-Jun signaling pathway [140], and it suppresses metastasis in gastric cancer and prevents migration and invasion by targeting NFкB/Wnt5a/JNK signaling [141]. GPx3 can inhibit the activation of NF-κB through the Erk pathway, leading to the suppression of the cell-cycle proteins B1 and G2/M and the inhibition of EMT by downregulating the Erk-NF-κB-SIP1 signaling axis [126,142].

#### 3.2.4. GPx4

The *GPX4* gene of the fourth selenium-containing GPx isoform is located on chromosome 19, specifically at band 19p13.3 in the human genome, and consists of seven exons that are expressed in three different forms, each with distinct transcription and translation starts [143]. GPx4 is a monomer composed of a thioredoxin (Trx) motif of four solvent-exposed alpha helices and seven beta strands, and it is present as three physiological isoforms: cytosolic (cGPx4), mitochondrial (mGPx4), and nuclear (nGPx4). *GPX4* can be expressed in a variety of tissues, with the highest content in the testes, affecting the development and function of sperm [144]. It should be noted that cGPx4 is expressed in most mammalian cells, whereas mGPx4 mostly appears in spermatoid cells, and nGPx4 is expressed in late spermatocytes [145,146].

**Figure 2:**
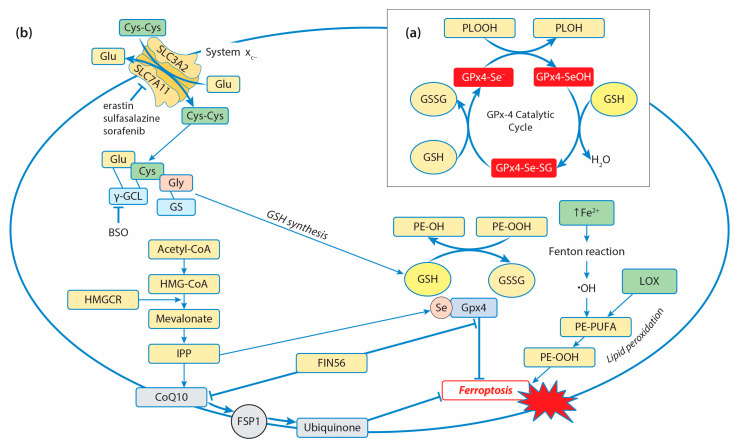
Role of GPx4 in the ferroptosis regulatory pathway: (**a**) *GPx4 catalytic cycle representation*. Under the reduction of lipid hydroperoxides (PLOOH) into their respective alcohols (PLOH), catalyzed by GPx4, GPx4 selenol (GPx4-SeH) is oxidized into selenic acid (GPx4-SeOH). Selenic acid is reduced to its active form, selenol, by a two-step reaction with GSH: The first GSH reacts with selenic acid to form a selenium–glutathione intermediate (GPx4-Se-SG), which is reduced by the second GSH to selenol. (**b**) *Regulation of ferroptosis by GPx4*. The conversion of polyunsaturated fatty acids (PUFAs) of phospholipids to peroxide PUFAs represents the initiation step to drive ferroptotic cell death. The phospholipid hydroperoxides (PE-OOH) are formed via non-enzymatic and enzymatic lipid peroxidation. The increased cytosolic labile iron pool (LIP) activates Fenton reactions and generates ^•^OH radicals, which initiate non-enzymatic lipid peroxidation with the formation of PE-OOH, and their levels can be raised by 15-lipoxygenase (LOX) activity. GPx4 catalyzes the reduction of toxic PE-OOH into nontoxic alcohol, ultimately inhibiting ferroptosis. The amino acid antiporter system xc- (composed of SLC3A2 and SLC7A11 subunits) mediates the exchange of intracellular glutamate and extracellular cystine, which is converted into cysteine, contributing to GSH synthesis. Inhibition of the key enzyme of GSH synthesis—γGCL (buthionine sulfoximine, BSO)—and SLC7A11 (erastin, sulfasalazine, sorafenib) leads to the activation of ferroptosis through GSH depletion. A selenocysteine residue is added to the catalytic center of GPx4 with the use of isopentenyl pyrophosphate (IPP), leading to GPx4 activation and ferroptosis inhibition. IPP is generated via the mevalonate pathway: acetyl-CoA is converted to 3-hydroxy-3-methylglutaryl-CoA (HMG-CoA). HMG-CoA is reduced by 3-hydroxy-3-methylglutaryl-CoA reductase (HMGCR) to mevalonate, which, in turn, is converted to IPP. IPP also generates coenzymeQ10 (ubiquinone, CoQ10), which is reduced to ubiquinol by ferroptosis-suppressor-protein 1 (FSP1), blocking lipid peroxidation. The ferroptotic inducer FIN56 works through a dual mechanism of depleting GPX4 protein and CoQ10 levels.

Among the GPx isoforms, which reduce small organic hydroperoxides, only GPx4 can reduce the large and complex lipid hydroperoxides and cholesterols, even when they are embedded in the biological membrane [147]. As a cofactor in reducing substrates, GPx4 uses the most preferred GSH, although it has the unique ability to utilize other protein thiols [148]. GPx4’s catalytic action is based on oxidation/reduction steps and involves the redox shuttling of the selenocysteine active site between an oxidized and a reduced state [149]. In the first phase, the reduction of the lipid peroxides to nontoxic lipid alcohols occurs due to the oxidation of the active site selenol (Se-H) to selenic acid (Se-OH) (Figure 2a).

During the second phase, a cosubstrate GSH is used to reduce the selenenic acid back to the active selenol and allow the oxidation/reduction process to be repeated. One molecule of GSH reacts with selenic acid to form a selenium–glutathione intermediate, while the second GSH reduces it to a selenol-releasing glutathione disulfide (GSSG), which is reduced by GR. It has been suggested that GPx4 might utilize either the “low-oxidation” (R-SeO-) or the “high-oxidation” (R-SeOO) cycle, depending on cellular conditions. GPx4 is considered to be the core regulator of ferroptosis since it reduces phospholipid hydroperoxides by catalyzing the conversion from R-OOH into R-OH, preventing iron-dependent lipid-reactive oxygen production and inhibiting ferroptosis (Figure 2b) [147,148]. Ferroptosis is a form of programmed cell death defined as an iron-catalyzed form of regulated necrosis, and it is driven by iron-dependent phospholipid peroxidation, featuring the accumulation of ROS and overproduction of lipid peroxidation [150].

The four ways of initiating ferroptosis are currently discussed [151]: Class I ferroptosis inducers work via the inhibition of cystine import by system xc- and driving depletion of GSH, class II ferroptosis inducers act by directly targeting and inactivating GPx4, class III ferroptosis inducers cause depletion of GPx4 and CoQ10 generated via the squalene synthase (SQS)–mevalonate pathway, and class IV ferroptosis inducers increase the cytosolic labile iron pool (LIP) or oxidize iron to induce lipid peroxidation [152]. Elevated ROS production and elevated iron requirements make cancer cells more susceptible to ferroptosis, and high levels of *GPX4* expression can be an inhibition factor in the development of ferroptosis. Indeed, in various types of cancer, its expression is generally higher than in normal tissues, including kidney chromophobe carcinoma, prostate adenocarcinoma, thyroid carcinoma, colon adenocarcinoma, kidney renal clear-cell carcinoma, cervical and endocervical cancer, lung adenocarcinoma, and rectal adenocarcinoma [146]. Numerous studies have shown that GPx4 inhibitors enhance sensitivity to chemotherapy, radiotherapy, and immunotherapy by inducing ferroptosis [144]. For example, enhanced ferroptosis as a result of GPx4 inhibition increases the sensitivity of colorectal cancer to oxaliplatin, non-small-cell lung cancer to lapatinib, and Epstein–Barr-virus-infected nasopharyngeal carcinoma to platinum [153,154,155]. Among the three known GPx4 isoforms, only the cytosolic isoform (cGPx4) is required for preventing ferroptosis. Direct and indirect inhibitors of GPx4 are used for the activation of ferroptosis. Some of them have been tested as a new approach in antitumor therapies in vitro. The drugs can be classified as follows: (1) Drugs directly or indirectly inhibiting system *x*c- (e.g., erastin, sorafenib, and sulfasalazine); (2) Drugs inhibiting GSH synthesis through the suppression of γGCL (e.g., buthionine sulfoximine, BSO); (3) Drugs inhibiting GPx4 (e.g., RSL3, withaferin A, and FIN56) [156]. Some of the chemotherapeutic drugs (e.g., cisplatin and altretamine) are able to promote ferroptosis through direct and indirect inhibition of GPx4 [157]. Cisplatin leads to GSH depletion and GPx4 inactivation, inducing both ferroptosis and apoptosis in A549 non-small-cell lung cancer (NSCLC) cells and colorectal carcinoma (HCT116 CRC) cells [158]. Altretamine (hexamethylmelamine) inhibits GPx4 and effectively kills U-2932 diffuse large B-cell lymphoma (DLBCL) cells in vitro [159]. The specific inducer of ferroptosis known as Ferroptosis-Inducer-56 (*FIN56*) was identified as an inducer that works through a dual mechanism of depleting GPx4 protein and mevalonate-pathway-derived coenzyme Q10 (CoQ10) [160]. Some signaling mechanisms are included in the regulation of *GPX4* expression and the modulation of ferroptosis in cancer cells. The upregulation of prostaglandin E receptor (PTGER3) weakens the epithelial–mesenchymal phenotype in triple-negative breast cancer and promotes ferroptosis both in vitro and in vivo by repressing *GPX4* expression [161]. On the other hand, the downregulation of PTGER3 inhibits ferroptosis by increasing *GPX4* expression and activating the PI3K-Akt pathway. In esophageal squamous-cell carcinoma (ESCC), the expression of *GPX4* was downregulated by the knockdown of aurora kinase A (AURKA), which led to the activation of ferroptosis and suppression of cancer progression. AURKA acts as a tumor-promoting gene and may serve as a potential target for ESCC treatment [162]. Similarly, inhibition of AURKA by using siRNA or miR-4715-3p reconstitution in gastric cancer suppressed *GPX4* and induced cell death [163]. In glioblastoma multiforme (GBM) cells, FOXP3, belonging to the forkhead box (FOX) family, was found to upregulate the transcription of *GPX4*, but it also attenuated the degradation of *GPX4* mRNA through the linc00857/miR-1290 axis, thereby suppressing ferroptosis and promoting proliferation [164].

#### 3.2.5. GPx5 and GPx6

The studies of the functions of GPx5, GPx6, GPx7, and GPx8 in cancer development are limited in comparison with those of Gpx1–Gpx4. GPx5 is a specifically epididymis-expressed enzyme containing Cys residues instead of Sec at its active site [165]. The *GPX5* gene is located on chromosome 6p22.1, 9075 bp in size, and contains six exons. The purified active mammalian GPx5 protein is a homotetramer consisting of subunits with a molecular weight of 25 kDa. GPx5 plays an important role in maintaining the microenvironment of the epididymis, protecting sperm from oxidative stress, and maintaining the integrity of the DNA structure [16].

Like *GPX5*, the *GPX6* gene is located on chromosome 6p22.1, with 12,498 bp, and contains five exons. GPx6 is closely homologous to GPx3; it is a homotetramer with 25 kDa subunits. In humans, GPx6 is a selenoprotein with Sec at the active center. In other mammals, selenocysteine cannot be synthesized due to the lack of selenocysteine insertion sequences in related genes, and GPx6 is a non-selenium protein replaced by Cys [94]. *GPX6* is mainly expressed in embryonic and olfactory organ epithelial cells and may be involved in the transmission and degradation of odor-related signals. GPx6 has not been purified, and its kinetics data are unavailable, resulting in minimal understanding of this GPx isoform. However, in a study on the inhibition of Hepa1-6 cell proliferation induced by oxidative stress, it was found that increased expression of the GPx6 protein may play a role in inhibiting oxidative stress [166]. It has also been shown that GPx5 and GPx6 were downregulated in the MDA-MB-231 human breast cancer cells compared with healthy MCF-10A breast cells [167].

#### 3.2.6. GPx7 and GPx8

GPx7 and GPx8 evolved from a common GPx4 ancestor [168]. Like GPx4, GPx7 and GPx8 are monomeric due to their lack of an oligomerization interface [169], and they have a molecular weight of 21 and 24 kDa, respectively. The human *GPX7* gene is located on chromosome 1p32.3, with 6681 bp, and contains four exons, while the *GPX8* gene is located on chromosome 5q11.2, 7127 bp in size, and contains four exons. In humans, they show 32 and 28% sequence identity to *GPX4*, respectively. Mammalian GPx7 and GPx8 are unique homologs, which are non-selenium-containing GPx isoforms (CysGPx) that localize to the ER. GPx7 is located in the ER lumen, while ER-anchored human GPx8 is a type II transmembrane protein, where the catalytically active cysteine is located in the ER lumen. After cutting N-terminal signaling, GPx7 is transferred from the ER to the Golgi apparatus along secretory pathways [170].

Both GPx7 and GPx8 have low GSH peroxidase activity. GPx7 does not contain domains bound to GSH, so it cannot participate in redox reactions with GSH. In response to oxidative stress, GPx7 can promote stress signal transduction through interaction with corresponding targeted proteins, such as the 78 kDa glucose regulatory protein (GRP78) and protein disulfide isomerase (PDI). Under oxidative stress, H_2_O_2_ catalyzes the formation of disulfide bonds between Cys57 and Cys86 on GPx7, prompts the transformation of GPx7 from reduced to oxidized form, and then oxidized GPx7 triggers the formation of disulfide bonds between Cys41 and Cys420 on GRP78, or between Cys53 and Cys56 on PDI, enhancing their activity [16]. GRP78 is one of the main chaperone proteins in the ER, and under unfolded protein stress conditions, it binds to unfolded or misfolded proteins and activates downstream ER stress sensors, including inositol-requiring enzyme 1α (IRE1), protein kinase RNA-like ER kinase (PERK), and activating transcription factor 6 (ATF6), to trigger a response to unfolded proteins and the refolding process of misfolded proteins [170]. Loss of GPx7 leads to impaired chaperone activity of GRP78 and to the accumulation of unfolded proteins, resulting in elevated oxidative stress [171]. The formation of intramolecular disulfide bonds during the maturation of many secreted proteins and membrane proteins requires not only the GRP78 chaperone protein but also the involvement of PDI and ER redox protein 1 (ERO1). Under oxidative stress, activated ERO1 generates disulfide bonds by consuming O_2_ in the presence of flavin cofactors, which are then passed to the protein for folding via PDI [172,173]. GPx7 can utilize the H_2_O_2_ produced by ERO1 to accelerate the oxidative folding process of proteins in vitro and in vivo and interact with the domain of PDI. Intramolecular cooperation between the two redox-active sites of PDI increases the activity of the ERO1α/GPx7/PDI triplet, thereby promoting protein-folding mechanisms [173]. Thus, GPx7 plays a unique role in maintaining redox homeostasis in response to oxidative stress. The dysregulation of *GPX7* may lead to some diseases, including cancer. It has been shown that the differential expression of *GPX7* is closely related to the occurrence and progression of multiple tumors. *GPX7* was found to be overexpressed in hepatocellular carcinoma tissues [174]. *GPX7* also exerted a tumor-suppressing function in gastric cancer and was silenced by promoter DNA methylation [175]. A bioinformatics study revealed that elevated *GPX7* is involved in the progression of glioma through several enriched pathways, including the cell-cycle pathway, focal adhesion pathway, and toll-like receptor pathway [176]. These findings provide key clues to further study the basic biology of *GPX7* in glioma. It has recently been found that *GPX7* expression is a potential prognostic biomarker in lower-grade glioma [177].

GPx8 has a similar function to GPx7 and can increase the activity of ERO1, promoting the oxidative folding of endoplasmic reticulum proteins [178]. GPx8 reduces oxidized PDI and prevents endoplasmic reticulum oxidoreductase 1alpha (ERO1α)-derived H_2_O_2_ leakage by regulating ERO1α [178]. A loss of GPx8 causes ER stress, leakage of ERO1α-derived H_2_O_2_ to the cytosol, and cell death. However, unlike GPx7, GPx8 has a transmembrane domain, which plays a key role in the regulation of Ca^2+^ signaling [179]. Therefore, GPx8 participates not only in the folding of proteins in the ER but also in the regulation of Ca^2+^ in the endoplasmic reticulum.

A correlation between GPx8 and poor prognosis has been reported in various cancer types. GPx8 can maintain the invasive mesenchymal-like phenotype of breast cancer cells through the IL-6/STAT3 axis [180]. GPx8 has been recognized as a prognostic marker for cancers such as primary glioma and gastric cancer [181,182]. In clear-cell renal cell carcinoma, *GPX8* silencing inhibits tumorigenesis by regulating nicotinamide N-methyltransferase (NNMT) [183]. These studies demonstrate the importance of aberrant expression of *GPX8* in carcinogenesis, which may serve as a potential target for cancer therapy. In a series of bioinformatics analyses, *GPX8* was identified as a key prognostic gene expressed in cancer-associated fibroblasts (CAFs), playing a crucial role in the tumor microenvironment of lung adenocarcinoma [184]. The high expression of *GPX8* was associated with poor patient survival and the formation of an immunosuppressive microenvironment.

## 4. Glutathione Transferases and Tumorigenesis

### 4.1. GST Family and Conjugation of Electrophiles to GSH

The family of GSTs catalyzes the conjugation of many endobiotic and xenobiotic electrophiles to GSH and plays central roles both in the biotransformation of xenobiotics and in the antioxidant defense system [19,20,185,186]. Three GST subfamilies are present in mammals: canonical soluble, mitochondrial, and membrane-associated enzymes. In humans, the mitochondrial GSH transferase is a singleton, whereas the membrane-associated proteins comprise six members, and the canonical family is encoded by 17 genes segregated into seven classes. The cytosolic GSTs (cGSTs) are divided into alpha, mu, pi, omega, theta, sigma, and zeta (A, M, P, O, T, S, Z) classes, whereas mitochondrial GSTs (mGSTs) include A, M, P, and kappa (K) classes. A novel subfamily designated MAPEG (membrane-associated proteins in eicosanoid and glutathione metabolism) includes members of widespread origin with diversified biological functions. Members of the MAPEG family include leukotriene C-4 synthase, 5-lipoxygenase-activating protein, prostaglandin E synthase, and microsomal glutathione S-transferases (MGST) 1, 2 and 3 [19]. The canonical or soluble GST enzymes include homodimer and heterodimer isoforms. Mitochondrial GST kappa (GSTK1-1) is located in both mitochondria and peroxisomes in human cells, and it is distinct from the cytosolic GSTs due to a putative cleavable N-terminal signal for mitochondrial translocation and a C-terminal signal sequence, Ala-Arg-Leu, for peroxisomal targeting [187]. Microsomal glutathione S-transferase 1 (MGST1), a representative of the MAPEG subfamily, is a homotrimeric protein with three glutathione (GSH)-binding sites, abundant in the ER and outer mitochondrial membranes [19].

All GSTs have a basic protein fold comprising two subunits with C-terminal and N-terminal domains. The N-terminal domain, consisting of four β-folds and three α-helices, includes a thioredoxin-like fold, β-α-β-α-β-β-α, where a β-β-α motif, known as the G-site, serves as the binding site for GSH through the γ-glutamyl unit. The G-site’s sequence similarity divides canonical GSTs into two subgroups: Tyrosine-type GSTs contain a Tyr residue (T- or P-class), which activates GSH [188], while the Ser/Cys-type GSTs (O-class) use Ser or Cys to form mixed disulfides with GSH [189]. These GSTs are more involved in redox reactions. The C-terminal domain, designated as the H-site, has an all-α-helical structure. The variability in H-site structure determines the substrate selectivity of various GST isozymes [19]. The H-site can non-specifically bind to a large variety of hydrophobic substrates with varying affinities, such as heme, bilirubin, dexamethasone, and polycyclic aromatic hydrocarbons [190]. The catalytic mechanism of the two sites involved in GSTs is as follows: (a) GSH binds to the G-site to form the strong nucleophilic thiolate anion; (b) The electrophilic substrate bound to the H-site reacts with the thiolate anion of GSH to form the GSH conjugate, which will be released via the C-terminus [191,192]. GSTs are important phase II detoxification enzymes involved in the detoxification of exogenous and endogenous substances [19]. The hydrophilic GSH conjugate (R-SG) formed intracellularly is excreted from the cell by the multidrug-resistance-associated protein (MRP). The major types of GST-catalyzed reactions include epoxide ring opening, nucleophilic aromatic substitution reactions, Michael addition of α,β-unsaturated aldehydes and ketones, isomerization, and peroxidase reactions [22,193].

Cytosolic GSTs catalyze the thiolysis of 4-nitrophenyl acetate; exhibit thiol transferase activity; reduce trinitroglycerin, dehydroascorbic acid, and monomethyl decanoic acid; and isomerize ketosteroids. GSTs have selenium-independent GPx activity and are able to reduce hydroperoxides of phospholipids and free fatty acids as well as cholesterol hydroperoxides [19]. The GSTA4-4 isoform possesses a higher affinity for 4-hydroxy-2-trans-nonenal (4-HNE), which is a potentially toxic stable end product of lipid peroxidation, a common denominator in stress-mediated signaling, and a pro-apoptotic second messenger altering cell-cycle signaling pathways in a concentration-dependent manner [194].

Cytosolic GSTs not only catalyze the conjugation of GSH to electrophilic substances (including electrophilic drug metabolites and endogenous electrophiles) and reduction of organic hydroperoxides but also take part in the regulation of cellular signaling pathways, such as the mitogen-activated protein (MAP) kinase pathway, via the inhibition of c-Jun N-terminal kinase 1 (JNK1) and apoptosis signal-regulating kinase 1 (ASK1) [20,192]. These GSTs are included in the protein’s post-translational modification by S-glutathionylation or deglutathionylation, and they make a great contribution to resistance to multiple chemotherapeutic drugs.

### 4.2. GST Polymorphisms in Cancer

Polymorphisms related to the GSTs’ genome cause changes in the molecular structure of the enzyme, affecting its stability and the variability of enzymatic activity and detoxification capacity, which may lead to various types of cancer [195]. Some data on GST gene polymorphisms are shown in Table 2.

M-class GSTs can prevent or repair damage to DNA by inactivating carcinogens and lipid peroxidation products. Deletion of the *GSTM1* gene can lead to inactivation of GSTM1-1, altering resistance to poisons and carcinogens, which can result in the loss of detoxification capacity and increase the risk of cancer development. Thus, the *GSTM1*-null genotype is associated with the risk of developing nasopharyngeal carcinoma and significantly shorter survival in patients with colorectal cancer [196,197]. Cytosolic GSTM1-1 and GSTT1-1 are especially significant in the biotransformation of polycyclic aromatic hydrocarbons, which can be detected in processed meat and cigarette smoke, which are well-recognized contributing factors to the development of colorectal cancer [198]. Deletion in the *GSTT1* gene results in a deficiency of GSTT1-1 activity (especially against the halogenated hydrocarbons and pesticides), affecting the detoxification capacity of the individual and increasing their susceptibility to carcinogenic compounds. A strong correlation was found between the *GSTT1*-null genotype and Philadelphia-negative chronic myeloid leukemia (Ph-ve CML), whereas the data for *GSTM1* polymorphisms indicate no role in the initial development of the disease [199]. At the same time, a relationship between deletion in the *GSTT1* and *GSTM1* genes and an increased risk of occurrence for many types of cancer (e.g., liver cancer, breast cancer, cervical cancer, head and neck cancer, esophageal cancer, oral cancer, lung cancer) has been shown [195].

Functions of the O-class glutathione transferases (GSTO1-1 and GSTO2-2) include thioltransferase and dehydroascorbate reductase activity as a consequence of cysteine’s presence in the active site [200]. GSTO1-1 is known for its significant role in the S-glutathionylation cycle due to its deglutathionylase and glutathionylase activity. GSTO2-2 has very high GSH-dependent dehydroascorbate reductase activity and is remarkably highly expressed in the testes [200]. An association was found between the GSTO1*C419A polymorphism (rs4925) and susceptibility to various cancers, including acute lymphoblastic leukemia, hepatocellular, breast, bile duct, non-small-cell lung, colon, and testicular cancers [201], while the GSTO2*A424G polymorphism (rs156697) is related to ovarian, breast, urinary bladder, and renal cell cancers [202,203]. It has been suggested that genetic variations caused by the *GSTO2* rs156697 polymorphism affect *GSTO2* dehydroascorbate reductase [201]. Reduced dehydroascorbate reductase activity in individuals with variant *GSTO2* alleles might result in deficient recycling mechanisms of vitamin C and accumulation of dehydroascorbate [204], contributing to the disruption of redox homeostasis. This might further significantly affect the antioxidant capacity in homozygous individuals, potentially contributing to the process of carcinogenesis in susceptible individuals. In this connection, the analysis of *GSTO2* genotypes (rs156697 and rs2297235) confirmed that the combined GSTO2*A/G*G/G and GSTO2*A/G*G/G genotype was associated with a significantly increased risk of testicular germ-cell cancer [201]. The P class also actively protects cells from carcinogens and electrophilic compounds. The *GSTP1* gene plays an important role in several cellular processes, including detoxification of electrophilic compounds, oxidative stress regulation, cell signaling, and carcinogenesis. It has been reported that GSTP1-1 inactivation is often observed in human cancers (e.g., liver cancer, breast cancer, prostate cancer, leukemia). Epigenetic modifications in the *GSTP1* gene can be recognized as biomarkers for the diagnosis of cancer in its early stages and, thus, for prophylaxis or treatment monitoring [195]. A meta-analysis conducted in 2024 found that the *GSTP1* Ile105Val gene polymorphism is significantly associated with susceptibility to acute myeloid leukemia, especially among the non-Asian population [205]. The genotype combination *GSTT1* (non-null)/*GSTP* (Ile/Val + Val/Val) has increased susceptibility to gallbladder cancer and may be considered as “at risk” genotype in North Indians [206]. In the case of the *GSTP1 A/G* gene polymorphism, an increased incidence of breast cancer was found in Asian women, while this relationship was not found in European or African women [207]. A meta-analysis conducted in 2023 showed that the *GSTP1* rs1695 polymorphism was significantly correlated with platinum-induced toxicities; it was concluded that personalized chemotherapy based on these polymorphisms could be considered for cancer patients in the future [208]. Among the various single-nucleotide polymorphisms (SNPs) of *GSTP1*, Ile105Val polymorphisms are the most widely studied. Based on an in silico study with ethacrynic acid [209], it has been demonstrated that the binding capacity of ethacrynic acid decreases with the Ile105Val mutation of *GSTP1*, indicating the changes in its anticancer activities. Cancer cells expressing *GSTP1* Val105 exhibit greater tolerance to ethacrynic acid-induced toxicity. It has been suggested that understanding the correlation between *GSTP1* Ile105Val polymorphisms and responses to GSTP1-1 inhibitor treatment would offer valuable insights for future drug development targeting *GSTP1* in cancer-related diseases.

*GSTP1* plays a major role in the metabolism of cisplatin and carboplatin in ovarian cancer cells, and it may be used as a target gene and response biomarker for platinum-based chemotherapy [213]. Both genetic and pharmacological inhibition in vivo were used to show that GSH conjugates of platinum are catalyzed by GSTP1-1 [214]. Cisplatin-induced nephrotoxicity could be diminished using GSH mimetics [215]. Polymorphisms within the *GSTP1* gene may especially alter enzyme activity and toxicities in patients receiving platinum-based chemotherapy. A meta-analysis conducted in 2022 showed that the *GSTP1* rs1695 polymorphism was significantly correlated with platinum-induced toxicities [208]; the study also revealed that rs1695 expression exhibited tissue-specific patterns and, thus, yielded opposite effects in different tissues. It was suggested that a personalized chemotherapy treatment based on these polymorphisms could be considered for cancer patients in the future.

Expression of GSTs, especially GSTP1-1, is increased in cancer. It was found that the expression of GST isozymes is upregulated in 60 human tumor cell lines at both the mRNA and protein levels, and GSTP1 is the most abundant isozyme in all of these cell lines [216]. Overexpression of *GSTP1* has been found in different types of cancer and may be involved in the development of resistance to chemotherapeutics in cancer cells, such as the resistance of ovarian cancer cells against carboplatin and cisplatin, breast cancer cells and prostate cancer cells against adriamycin, gastric cancer cells against 5-fluorouracil and cisplatin, and neurogliomas against cisplatin and irinotecan [22,217,218]. A novel mechanism of GSTP1-1’s action in the development of resistance to adriamycin in breast cancer cells has been established [219]. The high level of GSTP1-1 maintains the resistance of MCF-7 cells to ADR by promoting autophagy. It has been found that GSTP1-1 enhanced the autophagy levels in MCF-7/ADR cells by interacting with the p110α subunit of phosphatidylinositol-3-kinase (PI3K) and then inhibiting PI3K/Akt/mTOR activity. Proline123, leucine160, and glutamine163, which are located in the C-terminal domain of GSTP1-1, are essential for GSTP1-1 to interact with p110α, as well as the subsequent regulation of autophagy and drug resistance.

The promoter region of the *GSTP1* gene is usually affected by methylation, and changes in methylation status suppress normal gene expression, which may lead to weakening or loss of its detoxification and antioxidant functions [220]. In several types of cancer, the *GSTP1* gene is affected by hypermethylation. *GSTP1* is a major tissue biomarker that performs well in several types of malignancies, such as prostate, breast, and lung cancers, as well as hepatocellular carcinoma [220]. For example, the results of a meta-analysis substantiated the high specificity of promoter methylation of *GSTP1* in cell-free DNA (cfDNA) for the diagnosis of prostate cancer, and this could be used to more precisely evaluate the prognosis of patients with this type of cancer [221]. This may be helpful for the early detection of prostate cancer, but it must still be combined with traditional prostate-specific antigen (PSA) or other methylated genes to accomplish this goal. Recently, it was found that the promoter hypermethylation of the *GSTP1* gene, along with the *RARB* gene, which encodes retinoic acid receptor beta, is associated with breast cancer, older age, and postmenopausal Peruvian patients [222].

### 4.3. GSTs’ Chaperone Function in the Regulation of *Stress-Induced* Signaling Pathways

In addition to xenobiotic detoxification function, GSTs exhibit significant ligand-binding properties, and several GST isoenzymes have been shown to interact with stress kinases, controlling the cell signaling pathways responsible for stress response, cell proliferation, and apoptosis [20]. GSTP1-1 is involved in cell proliferation and apoptosis by regulating the phosphorylation of key signaling effectors, such as JNK and TNF receptor-associated factor 2 (TRAF2). GSTP1 functions as a chaperone to JNK, implying a role in cell survival and apoptosis as a member of the MAPK pathway. When cells are in a non-stress state, GSTP1 binds to JNK, inhibiting its activity; meanwhile, under stress conditions, GSTP1-1 dissociates from the complex and accumulates in oligomeric structures, resulting in the release and activation of JNK for subsequent phosphorylation of downstream targets regulating cell proliferation and apoptosis [223,224]. Another chaperone activity of GSTP1-1 inhibits the function of TRAF2, an upstream regulator of JNK, thereby blocking the MAPK/JNK signaling cascade at multiple steps [225]. The overexpression of GSTP1 suppressed TRAF2-induced activation of both JNK and p38 as well as attenuating autophosphorylation of ASK1 and inhibiting TRAF2-ASK1-induced apoptosis in HeLa human cervical cancer cells [225]. Conversely, the silencing of *GSTP1* led to the triggering of TRAF2-ASK1 association and hyperactivation of ASK1 and JNK. Nuclear mitotic apparatus protein 1 (NUMA1)’s transcript and protein levels were significantly upregulated in esophageal squamous-cell carcinoma patient samples, and its high expression predicated poor prognosis. It was revealed that NUMA1 interacted with GSTP1-1 and TRAF2, promoted the association of TRAF2 with GSTP1, and inhibited the interaction of TRAF2 and ASK1, regulating the sustained activation of JNK [226]. These findings suggest that NUMA1 plays an important role during the progression of esophageal squamous-cell carcinoma, and it functions by regulating the ASK1-MKK4-SAPK/JNK signaling pathway. Like GSTP1-1, GSTM1-1 binds to ASK1 and inhibits its activity. Under stress conditions, the GSTM1-1–ASK1 complex dissociates, causing the oligomerization of GSTM1-1 and the activation of ASK1, which subsequently activates the JNK and P38 pathways, leading to apoptosis [227]. Elevated expression of GSTM1-1 is associated with an impaired clinical response to therapies in a number of different types of cancer [20]. GSTA1-1 can also bind to and suppress the activation of JNK signaling via pro-inflammatory cytokines or oxidative stress [228]. GSTA1-1 negatively regulates the mTOR signaling pathway. Overexpression of *GSTA1* in patients with hepatocellular carcinoma has been found to indicate longer overall and disease-free survival and restrain the proliferation, migration, and invasion of liver cancer cells. GSTA1-1 may act as a protective factor through the suppression of tumorigenesis by targeting AMPK/mTOR due to increasing AMPK activity and inhibition of the mTOR pathway [229]. In SH-SY5Y human neuroblastoma cells, GSTO1-1 has been shown to interact (directly or in a complex) with Akt and MEK1/2. It was suggested that GSTO1-1 enzyme activity inhibits the activation of these two kinases to maintain basal levels. This possible regulation by GSTO1-1 is of interest, as both kinases have hundreds of potential downstream targets that are known to contribute to various cellular processes, including survival, growth, proliferation, and metabolism [230].

### 4.4. GST Inhibitors and Their Antitumor Action

Considering the significant role of GSTs in the processes of malignant growth, the search for their effective inhibitors is underway [231,232]. GST inhibitors are classified based on their binding activity and structure, and they may be grouped into inhibitors that can bind to the G- or H-site of GST proteins, glutathione peptidomimetics, and several natural compounds that have been identified as GST inhibitors [192].

Ethacrynic acid is both a substrate and a potent inhibitor (H-site binder) of GSTP, GSTA, and GSTM enzymes, with the most potent inhibition activity of GSTP1-1 [233] due to the α,β-unsaturated carbonyl group, which is capable of covalently binding to cysteine residues in the active site of the enzyme [234]. Ethacrynic acid exerts an antiproliferative effect on tumor cells and increases the cytotoxicity of several alkylating agents, such as melphalan, carmustine, mitomycin C, and nitrogen mustard; however, its strong diuretic properties and lack of isozyme specificity make it less favorable for clinical use as a modulator of anticancer drugs.

An additional H-site binder and another inhibitor of GSTP1-1 and other GSTs, 6-(7-nitro-2,1,3-benzoxadiazol-4-ylthio)hexanol (NBDHEX) possesses antiproliferative properties against various cancer cells, including leukemia, melanoma, osteosarcoma, and small-cell lung cancers, and it can induce apoptosis alone or in combination with other antitumor agents, e.g., cisplatin, doxorubicin, vincristine, methotrexate, and temozolomide [235,236]. The action of NBDHEX includes the formation of a spontaneous intermediate σ-complex with GSH, which binds tightly to GSTP1-1 and results in the loss of its GSH-conjugating activity as well as the ability to form complexes with JNK1 and TRAF2 [237]. NBDHEX has been found to be active against drug-resistant cell lines. For example, NBDHEX treatment inhibited GSTP1-1 activity, and co-administration of adriamycin and NBDHEX promoted apoptosis of adriamycin-resistant breast cancer cells [238]. The combination of NBDHEX and adriamycin significantly enhances the inhibition of tumor growth, offering new insights for breast cancer treatment.

Ezatiostat hydrochloride (Telintra, TLK199), a glutathione derivative, binds to and inhibits GSTP1-1 and disrupts the binding of GSTP1-1 to JNK, and it can stimulate the differentiation of primitive cells into mature monocytes, granulocytes, and erythrocytes and prevent the generation of ineffective bone marrow in myelodysplastic syndromes [239]. In addition to GSTP1-1 inhibition, TLK199 increases the expression of multidrug-resistance-associated protein 1 (MRP1) and can elevate the efficacy of chemotherapeutic drugs [240,241]. Telcyta (TER286, or canfosfamide) is a GSH analog prodrug that is activated by GSTP1-1 into a highly cytotoxic phosphorodiamidate, which spontaneously forms antiproliferative alkylating aziridinium species [242,243]. Telcyta has undergone phase II and phase III clinical trials [244,245] and has been estimated to have promising antitumor activity, patient tolerance, and relatively low toxicity in clinical testing for non-small-cell lung cancer, breast cancer, and ovarian cancer [246]. Currently, an antibody-directed enzyme prodrug therapy treatment based on Telcyta is being developed through protein engineering to improve the catalytic activity of human GST P1-1 [247].

## 5. Protein S-Glutathionylation in Cancer

In cancer cells, the regulation of redox-dependent processes is used as one of the key mechanisms in metabolic reprogramming, which reflects the modification of metabolism to support the increased energy demand due to continuous growth and rapid proliferation. The changes in protein functions and enzyme activities through post-translational modification of the reactive thiol (-SH) group of Cys residues by S-glutathionylation are the most frequent methods of protein regulation in cancer cells. S-glutathionylation is reversible and can act as a regulatory switch to interconvert inactive and active forms of proteins, thereby mediating cell signaling and redox homeostasis [248,249,250].

**Figure 3:**
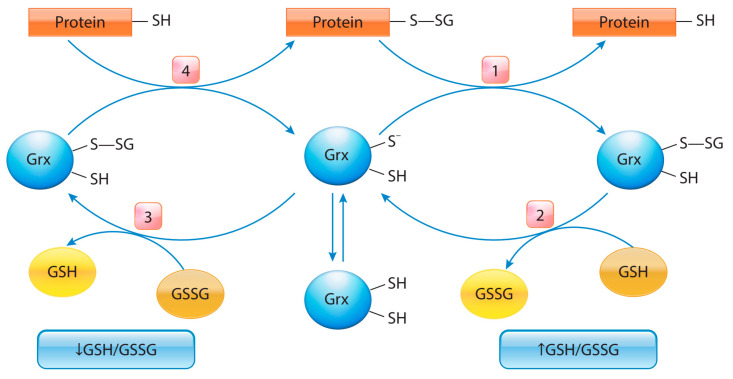
Glutaredoxin’s catalytic mechanism is dependent on the GSH/GSSH ratio: Under an increase in GSH/GSSG, Grx can catalyze the deglutathionylation of proteins. The glutathionylated sulfur moiety of the protein–SSG is attacked by the thiolate anion of the enzyme (Grx-S-), forming the covalent enzyme intermediate (GRx–SSG) and releasing the reduced protein–SH as the first product (1). The second rate-determining step involves the reduction of Grx–SSG by GSH to produce glutathione disulfide (GSSG) as the second product, recycling the reduced enzyme (Grx–S-) (2). Under conditions of decreased GSH/GSSG ratio, Grx can catalyze the S-glutathionylation of proteins. The S-glutathionylated Grx (Grx–SSG), formed in reaction with GSSG (3), reacts with a protein to create S-glutathionylated protein (protein–SSG) (4).

The pKa value of the cysteine thiol group is determined by the structure of its microenvironment and can vary significantly (from 3.5 to >12). Usually, at physiological pH (7.0–7.4), the value of pKa is ~8.5. A decrease in pKa can result from the stabilization of thiolate anions (Pr-S^−^) by the electron-acceptor groups or a neighboring positive charge. Conversely, the pKa value of thiolate increases in the presence of negatively charged groups or in the hydrophobic protein environment [248]. For instance, the pKa of the SH group decreases (usually to 5.0–7.0) in the immediate vicinity of basic amino acid residues (His, Lys, and Arg), whereas at physiological pH, the sulfhydryl groups dissociate. The formed thiolate anions are efficient nucleophiles whose reactivity toward electrophilic targets increases dramatically [251]. Non-enzymatic reactions of S-glutathionylation proceed via the thiol–disulfide exchange between protein thiol (Pr-SH) and GSSG [252], and they depend upon the GSH/GSSG ratio within the cell or occur via the reaction of GSH with an oxidized thiol derivative such as S-nitrosyl (-SNO), thiyl radicals (-S^•^), or sulfenic acid (-SOH) [253]. Nevertheless, GSTs can effectively reduce the pKa of the cysteine thiol, creating a more reactive nucleophilic thiolate anion [254]. Several GST isoenzymes have been reported to facilitate S-glutathionylation reactions. The leading role belongs to GSTP1-1, whose expression is a highly prognostic marker in a wide range of tumors [23,192]. Antioxidant proteins—e.g., redoxins, Trx [255], and sulfiredoxin (Srx) [256]—catalyze deglutathionylation, whereas Grx isoenzymes catalyze both S-glutathionylation and deglutathionylation reactions and are controlled by the GSH/GSSG ratio [257]. Deglutathionylation by Grx occurs through a thiol–disulfide exchange reaction [258], which leads to the formation of oxidized Grx. Reduced Grx is regenerated by using GSH as the reducing equivalent. When the GSH/GSSG ratio decreases, and the H_2_O_2_ content rises, Grx2 functions as a glutathionylation enzyme (e.g., toward respiratory complex I), whereas at a high GSH/GSSG ratio and low H_2_O_2_ concentration, it exhibits deglutathionylation activity [259] (Figure 3).

The interplay between Grx-catalyzed S-glutathionylation/deglutathionylation and cellular redox status may represent an adaptation to ensure that S-glutathionylation reactions will not be reversed as long as oxidative stress persists [260,261]. It has been suggested that the deglutathionylation activity of Grx is only functional when oxidative stress is removed [262]. Oxidative stress results in low GSH and high GSSG, both of which have an inhibitory effect on the deglutathionylating activity of Grx. Grx1 also regulates redox signal transduction and repairs protein oxidation by reversing S-glutathionylation. A significant correlation has been revealed between the expression of the Grx1 protein and stages I, II, and III of colorectal cancer [263]. The enhanced expression of not only Grx1 and Grx2 but also Grx3 has been detected in tumors [258]. The increase in Grx3 expression was associated with several solid tumors, including an increase in proliferation in colon and lung cancers [264], migration and invasion in oral squamous-cell carcinoma [265], and growth and metastasis in nasopharyngeal carcinoma [266]. Srx plays a preferential role in the deglutathionylation of certain proteins, such as Prx1, actin, and protein tyrosine phosphatase 1B (PTP1B) [256], possibly due to its higher affinity to such proteins compared to Grx. Unlike Grx, Srx is probably not inactivated by oxidative stress, as evident from its ability to diminish overall S-glutathionylation under oxidative stress [267]. Srx exclusively reduces over-oxidized typical 2-Cys Prx, and the Srx-Prx system plays a critical role in carcinogenesis by modulating cell signaling pathways involved in cell proliferation, migration, and metastasis that can define the Srx-Prx system as a future therapeutic target in human cancer [268].

It was shown that, in T47D breast cancer epithelial cells, deglutathionylation can be catalyzed by the glutathione transferase isoform GSTO1-1 [269,270]. GSTO1 is structurally similar to Grx, as it contains the Trx-like fold and GSH-binding site that can form a disulfide bond with GSH through the conserved Cys32 residue of the active site. In contrast, other GST isoforms (e.g., GSTA, GSTM, GSTP, GSTT, GSTS, and GSTZ) have Tyr or Ser as catalytic residues. Similar to Grx, GSTO1-1 catalyzes deglutathionylation in two steps: Cys32 of the GSTO1-1 active site interacts with Pr-SSG, resulting in the generation of reduced Pr-SH and the mixed disulfide GSTO1-1-Cys32-SG, which is further deglutathionylated with GSH through the formation of GSSG and functionally active GSTO1-1. 

**Figure 4:**
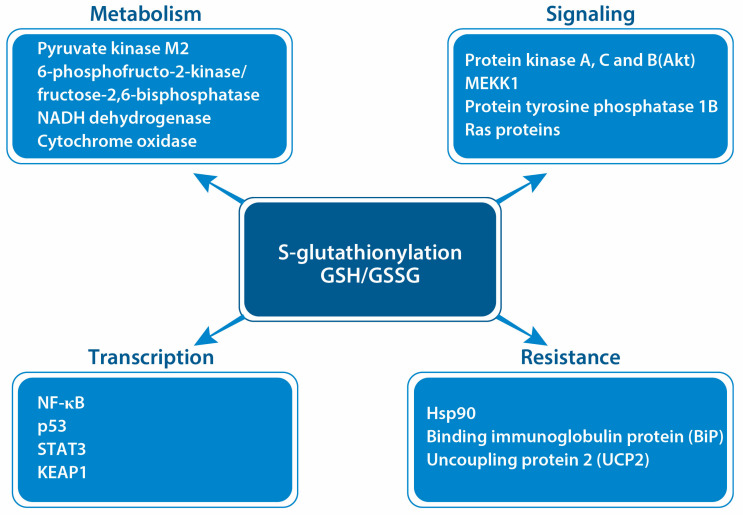
Some key targets in the regulation of cancer cell viability by S-glutathionylation: S-glutathionylation may cause changes in cellular metabolism via suppression of ATP production (inhibiting NADH dehydrogenase and cytochrome oxidase) and PPP promotion (inhibiting pyruvate kinase M2 and the bifunctional enzyme 6-phosphofructo-2-kinase/fructose-2,6-bisphosphatase). S-glutathionylation modulates cellular signaling pathways by changing the activity of protein kinases (protein kinases A, C, and B; MKK1), protein phosphatases (protein tyrosine phosphatase 1B), and Ras proteins, as well as regulating cell survival through changes in the activity of transcription factors (NF-κB, p53, STAT3, and Nrf2). S-glutathionylation also influences cancer cells’ viability through the regulation of their resistance to anticancer drugs (Hsp90, BiP, UCP2).

The increased level of GSH synthesis and expression of GSTP1 in most types of cancer cells, along with a reversed pH gradient (i.e., a lower extracellular pH of ~6.7–7.1 and a higher intracellular pH of 7.4) [271,272], may influence the rate of S-glutathionylation in the tumors. S-glutathionylation is involved in all major functional categories of cellular processes (Figure 4), including metabolism (e.g., glycolysis [273,274]), signal transduction (G-protein, kinase, and phosphatase [275,276,277]), development of resistance to anticancer drugs (heat shock protein 90 (Hsp90) [278]), and transcription regulation (e.g., p53 [279]).

S-glutathionylation targets an extremely broad range of proteins involved in all aspects of cancer cell activity. Thus, glutathionylation inhibits energy metabolism enzymes, including NADH dehydrogenase, cytochrome oxidase, ATPase, pyruvate dehydrogenase complex E2, and glyceraldehyde 3-phosphate dehydrogenase (GAPDH) [74]. By inhibiting pyruvate kinase M2 and the bifunctional enzyme 6-phosphofructo-2-kinase/fructose-2,6-bisphosphatase, which generates fructose 2,6-bisphosphate, S-glutathionylation promotes the PPP and facilitates the formation of NADPH(H^+^), which increases the GSH level by increasing the activity of GR [280]. Both caspase-3 (an important mediator of apoptosis) and its precursor procaspase-3 undergo S-glutathionylation. S-glutathionylation of procaspase-3 inhibits its capacity for proteolytic activation. The p17 subunit of caspase-3 is S-glutathionylated at Cys135, located in the active site, which affects its access to the substrate and suppresses enzyme activity [281].

Cellular signal pathways in cancer cells are modulated by S-glutathionylation at different levels. S-glutathionylation changes the activity of protein kinases A, C, and B. S-glutathionylation of PKA (Cys199) renders the kinase inactive, owing to steric hindrance decreasing its affinity for the substrate [282]. Its inactivation in the oxidative environment leads to a potential decline in proliferative signals and, thus, is an important point for anticancer signaling. The isoforms of PKC play a critical role in tumor proliferation, invasion, oncogenesis, and metastasis [283]. Overexpression of the PKC gene is associated with tumor growth due to the synergistic activation of several signaling pathways controlling cell survival and proliferation, including the NF-κB, Stat3, PI3K/Akt, and ERK pathways [284,285]. PKC isoforms (α, β, γ, ε, ζ) are inactivated by oxidative S-glutathionylation, as demonstrated using diamide and GSH [286]. PKB, also known as Akt, is also subject to regulation by S-glutathionylation at multiple levels. Usually, S-glutathionylation of Akt leads to its deactivation and inhibition of its downstream pathways. GSTO1-1 has been implicated in preventing the activation of Akt in SH-SY5Y cells, which suggests a possible role of S-glutathionylation [230]. S-glutathionylation has been shown to inactivate the phosphatases of the PI3-kinase-Akt pathway, which plays a critical role in cell survival and proliferation [287]. S-glutathionylation modulates intracellular signaling pathways by altering the activity of proteins—in particular, MEKK1 (mitogen-activated protein kinase kinase 1), protein tyrosine phosphatase 1B, and Ras proteins. S-glutathionylation of H-Ras at Cys118 modulates its intrinsic GTPase activity, eliciting activation of the downstream p38 and Akt [275].

The effect of S-glutathionylation is related to the regulation of cancer cells’ resistance to anticancer drugs. Hsp90 is a ubiquitous ATP-dependent chaperone that interacts with numerous proteins to regulate multiple cellular processes, especially during cell proliferation and cell-cycle progression. Hsp90 exists at high levels in tumor cells and tissues, serves as a prognostic biomarker or therapeutic target in cancers, and has also been implicated in conferring chemoresistance in various cancer cell types [288,289]. Cellular degradation of Hsp90 is dependent on the ubiquitin proteasomal pathway, and S-glutathionylation (Cys 366 and Cys 412) might play a crucial role in the process with the loss of ATPase activity and further structural changes in Hsp90 due to proteasomal degradation [290]. Thus, oxidative stress-inducing S-glutathionylation might prove to be a useful complimentary intervention to circumvent Hsp90-based resistance to chemotherapeutic drugs. 

Multiple myeloma cells’ resistance to bortezomib, a proteasome inhibitor, is associated with the S-glutathionylation of the binding immunoglobulin protein (BiP), with Cys41-SSG being important for ATPase and Cys420-SSG for foldase [291]. Enhanced levels of GSTP1-1 support S-glutathionylation in resistant cells. While ATPase activity is dampened as a result of glutathionylation, the foldase activity is enhanced, which results in refolding of unfolded proteins at a higher rate, ultimately nullifying the proteasome-inhibitory effect of bortezomib. Thus, S-glutathionylation of BiP confers pro-survival advantages and represents a novel mechanism of drug resistance in MM cells.

Overexpression of mitochondrial uncoupling protein 2 (UCP2), which results in increased proton leakage from the mitochondria and helps the cells to control ROS, is connected with resistance of drug-resistant leukemia cells to chemotherapeutic drugs. S-glutathionylation deactivates proton leakage through UCP2 in resistant human acute promyelocytic leukemia MX2 cells and sensitizes them to menadione and doxorubicin [292]. S-glutathionylation is the redox-dependent regulator of transcription factors and gene expression. The p53 tumor-suppressor protein is a versatile, redox-dependent transcription factor whose functional inactivation triggers oncogenic events and facilitates the emergence of unstable genomes in 50% of all human cancers; it is functionally inactivated by S-glutathionylation at Cys-141 during oxidative and DNA-damaging treatments [279]. High expression of NF-κB is correlated with shorter overall survival in patients with non-small-cell lung cancer, suggesting a tumor-promoting function for NF-κB. In response to oxidants, S-glutathionylation of NF-κB caused negative regulation by interfering with the DNA-binding activities of NF-κB subunits [293], suggesting that S-glutathionylation is an important factor in the regulation of NF-κB in clinical results for non-small-cell lung cancer cells, where NF-κB levels are associated with unfavorable prognosis. STAT3 is a transcription factor that is constitutively activated in a variety of cancers and plays a critical role in the inhibition of apoptosis; its deregulation is often associated with the development and progression of many solid and hematological tumors, as well as with drug resistance. Under oxidative conditions, STAT3 activity is regulated by S-glutathionylation [294]. Thus, sesquiterpene lactone cynaropicrin induces a rapid drop in intracellular GSH levels, thereby triggering S-glutathionylation of STAT3 and the suppression of anti-apoptotic genes, Bcl-2, and survivin in DU145 human prostate adenocarcinoma cells, leading to cell death through apoptosis and potentiation of the cytotoxic effects of cisplatin and docetaxel [295].

S-glutathionylation apparently may be used as a redox-dependent instrument in Nrf2 feedback regulation through cysteine residues of KEAP1. The increased levels of ROS cause the disruption of the KEAP1–Nrf2 complex and promotion of Nrf2’s translocation to the nucleus, which leads to enhanced expression of antioxidant and detoxification genes, including γGCL, a key enzyme of GSH synthesis [296,297]. The enhancement of GSH production in cancer cells may increase the S-glutathionylation level, causing KEAP1 modification through S-glutathionylation [298] and intensification of Nrf2 activation, which supports the increase in reductive stress. Reductive stress is defined as a condition characterized by excess accumulation of reducing equivalents (NADH, NADPH, and GSH), surpassing the activity of endogenous oxidoreductases [299]. The pool of reductive equivalents under reductive stress may not only reverse the oxidative stress but also lead to its activation [299,300]. Thus, reductive stress lowers cellular ROS levels below their physiological levels, disrupting their signaling functions. In contrast, reductive stress may promote the production of ROS, as redox couples can reduce O_2_ to ^•^O_2_^−^ in an oxygen environment [301,302,303]. Reductive stress can lead to the disruption of mitochondrial homeostasis, decrease metabolism, influence resistance to anticancer therapies, and alter the formation of disulfide bonds in proteins, leading to the activation of unfolded protein response (UPR)/ER stress [301]. The balance of oxidative/reductive stresses reflects the work of the redox pendulum in cellular redox status and the maximum changes that are connected with the extent of pathological damage. S-glutathionylation may serve as a modulator of this balance with regard to changing the GSH/GSSG state.

## 6. Conclusions

Numerous data and recent updates demonstrate the significance of GSH and the key GSH-related enzymes in the regulation of tumor cell viability, the initiation of tumor development, its progression, and drug resistance. The high level of GSH synthesis in different cancer types depends not only on the increasing expression of the key enzymes of the γ-glutamyl cycle but also on the changes in the transport velocity of its precursor amino acids. Nevertheless, the mechanisms controlling glutathione synthesis in malignant tumors are still poorly understood. As a strategy to counteract cancer progression and therapy resistance, it is promising to search for means of GSH depletion through the inhibition of key enzymes and/or precursors of its synthesis. The major GSH-related enzymes, i.e., the GPx and GST superfamilies, are the important links in the regulation of redox-dependent processes, which are used as key mechanisms in metabolic reprogramming in cancer and reflects the modification of metabolism to support the increased energy needed for continuous growth and rapid proliferation. The ability of GPxs to reduce hydroperoxides is used for cellular viability for multiple purposes including defense against oxidative challenge, redox regulation, and biosynthesis of important constituents. Expression aberrations and polymorphisms of different *GPX* genes and, consequently, their dual role in cancer call for future research in regulating complex *GPX*-dependent pathways for effective chemotherapy. GSTs not only catalyze the conjugation of GSH to electrophilic substances (including electrophilic drug metabolites and endogenous electrophiles) and the reduction of organic hydroperoxides but also take part in the regulation of cellular signaling pathways. By catalyzing the S-glutathionylation of key target proteins, GSTs are involved in the regulation of major cellular processes including metabolism (e.g., glycolysis and the PPP), signal transduction, transcription regulation, and the development of resistance to anticancer drugs. Taking into account the polymorphism of GST genes in different cancer types, the search for new effective GST inhibitors seems to be a promising direction in cancer chemotherapy.

## Figures and Tables

**Table 1:** Dual role of GPx1 in human cancers.

	Human Cell Line	Expression (Tumor vs. Normal)	Roles in Cancer	Target Action	Reference
Breast cancer	T47D	----	Tumor promoter	GPX1 overexpression inhibits doxorubicin-induced apoptosis	[106]
	MDA-MB-231, MDA-MB-468, Hs578T, BT-549	Up (mRNA, protein)	Tumor promoter	GPX1 expression promotes migration and invasion	[111]
	MDA-MB-231	----	Tumor promoter	GPx1 silencing increases TNF-α-induced apoptosis	[112]
Kidney cancer	A-498, ACHN, 786-O, CAKI-1	Up (protein)	Tumor promoter	GPX1 knockdown inhibits proliferation and clonogenic capacity	[113]
Glioma	Glioma stem cells U87, SU-2	Up (mRNA, protein)	Tumor promoter	Increased GPX1 expression decreases ROS levels and increases radioresistance	[114]
Lung cancer	A549, H1975, H460, H1650, GLC-82, H1993, H2170, Spc-a1, H1299	Up (protein, in cisplatin-resistant cell lines)	Tumor promoter	GPX1 overexpression inhibits ROS accumulation and leads to cisplatin resistance	[115]
Gastric cancer	SNU-1, -5, -16, -216,-484,- 601, -620, -638, -668, -719 cells	Down (mRNA, protein)	Tumor suppressor	Decreased GPX1 expression predicts aggressiveness, lymphatic invasion, and poor survival	[116]
Pancreatic cancer	MiaPaCa-2, SW1990, PANC-1	----	Tumor suppressor	GPX1 overexpression sensitizes cells to starvation-induced cell death via the activation of caspase-dependent apoptosis	[117]

**Table 2:** Some GST gene polymorphisms in cancer.

CST Class	GST Polymorphism	Cancer Type	Related Mechanism	References
GSTM	*GSTM1*-null	Nasopharyngeal cancer Colorectal cancer	Affects the risk of developing nasopharyngeal carcinoma in the Chinese population Associated with the risk of developing colorectal cancer and shorter survival in colorectal cancer patients	[196,197,198]
GSTT	*GSTT1*-null	Colorectal cancer Leukemia	Affects the risk of developing colorectal cancer Affects the risk of developing Philadelphia-negative chronic myeloid leukemia (Ph-ve CML)	[198,199]
GSTO	GSTO2*A/G*G/G and GSTO2*A/G*G/G	Testicular cancer	Associated with an increased risk of testicular germ-cell cancer	[200,201,202]
GSTP	*GSTP1* Ile105Val *GSTP1* rs1695 A>G GSTP1-rs1695 *GSTP1* rs4147581 GSTP1*Val rs1695 + GSTP1*Val rs1138272	Leukemia Breast cancer Lung cancer Liver cancer Prostate cancer	Affects the risk of developing acute myeloid leukemia Increases incidence of breast cancer in Asian women Affects the risk of developing lung cancer Prognostic marker for hepatocellular carcinoma Affects the risk of developing prostate cancer	[203,204,205,206,207,208,209,210,211,212]

## References

[1] Vázquez-Meza H., Vilchis-Landeros M.M., Vázquez-Carrada M., Uribe-Ramírez D., Matuz-Mares D. (2023). Cellular Compartmentalization, Glutathione Transport and Its Relevance in Some Pathologies. Antioxidants.

[2] Chen T.-H., Wang H.-C., Chang C.-J., Lee S.-Y. (2024). Mitochondrial Glutathione in Cellular Redox Homeostasis and Disease Manifestation. Int. J. Mol. Sci..

[3] Georgiou-Siafis S.K., Tsiftsoglou A.S. (2023). The Key Role of GSH in Keeping the Redox Balance in Mammalian Cells: Mechanisms and Significance of GSH in Detoxification via Formation of Conjugates. Antioxidants.

[4] Kennedy L., Sandhu J.K., Harper M.-E., Cuperlovic-Culf M. (2020). Role of Glutathione in Cancer: From Mechanisms to Therapies. Biomolecules.

[5] Kuehne A., Emmert H., Soehle J., Winnefeld M., Fischer F., Wenck H., Gallinat S., Terstegen L., Lucius R., Hildebrand J. (2015). Acute Activation of Oxidative Pentose Phosphate Pathway as First-Line Response to Oxidative Stress in Human Skin Cells. Mol. Cell.

[6] Gamcsik M.P., Kasibhatla M.S., Teeter S.D., Colvin O.M. (2012). Glutathione Levels in Human Tumors. Biomarkers.

[7] Hayes J.D., Dinkova-Kostova A.T., Tew K.D. (2020). Oxidative Stress in Cancer. Cancer Cell.

[8] Wang Y., Qi H., Liu Y., Duan C., Liu X., Xia T., Chen D., Piao H.-L., Liu H.-X. (2021). The Double-Edged Roles of ROS in Cancer Prevention and Therapy. Theranostics.

[9] O’Brien M.L., Tew K.D. (1996). Glutathione and Related Enzymes in Multidrug Resistance. Eur. J. Cancer.

[10] Valenti G.E., Tasso B., Traverso N., Domenicotti C., Marengo B. (2023). Glutathione in Cancer Progression and Chemoresistance: An Update. Red. Exp. Med..

[11] Jones C.M., Lawrence A., Wardman P., Burkitt M.J. (2002). Electron Paramagnetic Resonance Spin Trapping Investigation into the Kinetics of Glutathione Oxidation by the Superoxide Radical: Re-Evaluation of the Rate Constant. Free Radic. Biol. Med..

[12] Kirsch M., Lehnig M., Korth H.G., Sustmann R., de Groot H. (2001). Inhibition of peroxynitrite-induced nitration of tyrosine by glutathione in the presence of carbon dioxide through both radical repair and peroxynitrate formation. Chemistry.

[13] Winterbourn C.C., Metodiewa D. (1999). Reactivity of Biologically Important Thiol Compounds with Superoxide and Hydrogen Peroxide. Free Radic. Biol. Med..

[14] Cassier-Chauvat C., Marceau F., Farci S., Ouchane S., Chauvat F. (2023). The Glutathione System: A Journey from Cyanobacteria to Higher Eukaryotes. Antioxidants.

[15] Xing F., Hu Q., Qin Y., Xu J., Zhang B., Yu X., Wang W. (2022). The Relationship of Redox with Hallmarks of Cancer: The Importance of Homeostasis and Context. Front. Oncol..

[16] Pei J., Pan X., Wei G., Hua Y. (2023). Research Progress of Glutathione Peroxidase Family (GPX) in Redoxidation. Front. Pharmacol..

[17] Ferreira R.R., Carvalho R.V., Coelho L.L., De Souza Gonzaga B.M., Da Gloria Bonecini-Almeida M., Garzoni L.R., Araujo-Jorge T.C. (2024). Current Understanding of Human Polymorphism in Selenoprotein Genes: A Review of Its Significance as a Risk Biomarker. Int. J. Mol. Sci..

[18] Zhao Y., Wang H., Zhou J.-D., Shao Q. (2022). Glutathione Peroxidase GPX1 and Its Dichotomous Roles in Cancer. Cancers.

[19] Mannervik B., Morgenstern R. (2024). Glutathione Transferases. Comprehensive Toxicology.

[20] Mazari A.M.A., Zhang L., Ye Z.-W., Zhang J., Tew K.D., Townsend D.M. (2023). The Multifaceted Role of Glutathione S-Transferases in Health and Disease. Biomolecules.

[21] Townsend D.M., Shen H., Staros A.L., Gaté L., Tew K.D. (2002). Efficacy of a glutathione S-transferase pi-activated prodrug in platinum-resistant ovarian cancer cells. Mol. Cancer Ther..

[22] Lv N., Huang C., Huang H., Dong Z., Chen X., Lu C., Zhang Y. (2023). Overexpression of Glutathione S-Transferases in Human Diseases: Drug Targets and Therapeutic Implications. Antioxidants.

[23] Townsend D.M., Manevich Y., He L., Hutchens S., Pazoles C.J., Tew K.D. (2009). Novel Role for Glutathione S-Transferase π. J. Biol. Chem..

[24] Meister A., Anderson M.E. (1983). GLUTATHIONE. Ann. Rev. Biochem..

[25] Anderson M.E. (2022). Assay of the Enzymes of Glutathione Biosynthesis. Anal. Biochem..

[26] Lien E.C., Lyssiotis C.A., Juvekar A., Hu H., Asara J.M., Cantley L.C., Toker A. (2016). Glutathione Biosynthesis Is a Metabolic Vulnerability in PI(3)K/Akt-Driven Breast Cancer. Nat. Cell Biol..

[27] Lu S.C. (2009). Regulation of Glutathione Synthesis. Mol. Aspects Med..

[28] Lapenna D. (2023). Glutathione and Glutathione-Dependent Enzymes: From Biochemistry to Gerontology and Successful Aging. Ageing Res. Rev..

[29] Ristoff E., Larsson A. (2007). Inborn Errors in the Metabolism of Glutathione. Orphanet. J. Rare Dis..

[30] Almusafri F., Elamin H.E., Khalaf T., Ali A., Ben-Omran T., El-Hattab A.W. (2017). Clinical and Molecular Characterization of 6 Children with Glutamate-Cysteine Ligase Deficiency Causing Hemolytic Anemia. Blood Cells Mol. Dis..

[31] Da D., Pan Z., Zhang L., Dang Y., Dang C., Huang Y., Shi D., Li H. (2023). Glutamate-Cysteine Ligase Catalytic and Its Modifier Function as Novel Immunotargets in Gastric Adenocarcinoma. Asian J. Surg..

[32] Zhang L., Tang M., Tao X., Shao Q., Thomas V., Shimizu S., Kasano M., Ishikawa Y., Inukai T., Nomura D.K. (2023). Covalent Targeting of Glutamate Cysteine Ligase to Inhibit Glutathione Synthesis. ChemBioChem.

[33] Xue Z., Nuerrula Y., Sitiwaerdi Y., Eli M. (2024). Nuclear Factor Erythroid 2-Related Factor 2 Promotes Radioresistance by Regulating Glutamate-Cysteine Ligase Modifier Subunit and Its Unique Immunoinvasive Pattern. Biomol. Biomed..

[34] Hiyama N., Ando T., Maemura K., Sakatani T., Amano Y., Watanabe K., Kage H., Yatomi Y., Nagase T., Nakajima J. (2018). Glutamate-Cysteine Ligase Catalytic Subunit Is Associated with Cisplatin Resistance in Lung Adenocarcinoma. Jpn. J. Clin. Oncol..

[35] Luo L., Zhang Z., Weng Y., Zeng J. (2022). Ferroptosis-Related Gene GCLC Is a Novel Prognostic Molecular and Correlates with Immune Infiltrates in Lung Adenocarcinoma. Cells.

[36] Dequanter D., Van De Velde M., Bar I., Nuyens V., Rousseau A.F., Nagy N., Vanhamme L., Vanhaeverbeek M., Brohée D., Delrée P. (2016). Nuclear Localization of Glutamate-Cysteine Ligase Is Associated with Proliferation in Head and Neck Squamous Cell Carcinoma. Oncol. Lett..

[37] Liu C., Hua K.T., Li K., Kao H., Hong R., Ko J., Hsiao M., Kuo M.-L., Tan C.-T. (2017). Histone Methyltransferase G9A Drives Chemotherapy Resistance by Regulating the Glutamate–Cysteine Ligase Catalytic Subunit in Head and Neck Squamous Cell Carcinoma. Mol. Cancer Ther..

[38] Bykanova M., Solodilova M., Azarova I., Klyosova E., Бушуева О.Ю., Polonikova A., Churnosov M., Polonikov A. (2022). Genetic Variation at the Catalytic Subunit of Glutamate Cysteine Ligase Contributes to the Susceptibility to Sporadic Colorectal Cancer: A Pilot Study. Mol. Biol. Rep..

[39] Li M., Zhang Z., Yuan J., Zhang Y., Jin X. (2014). Altered Glutamate Cysteine Ligase Expression and Activity in Renal Cell Carcinoma. Biomed. Rep..

[40] Koyani C.N., Kitz K., Rossmann C., Bernhart E., Huber E., Trummer C., Windischhofer W., Sattler W., Malle E. (2016). Activation of the MAPK/Akt/Nrf2-Egr1/HO-1-GCLc Axis Protects MG-63 Osteosarcoma Cells against 15d-PGJ2-Mediated Cell Death. Biochem. Pharmacol..

[41] Laoukili J., Constantinides A., Wassenaar E., Elias S.G., Raats D., Van Schelven S.J., Van Wettum J., Volckmann R., Koster J., Huitema A.D.R. (2022). Peritoneal Metastases from Colorectal Cancer Belong to Consensus Molecular Subtype 4 and Are Sensitised to Oxaliplatin by Inhibiting Reducing Capacity. Br. J. Cancer.

[42] Sun J., Zhou C., Ma Q., Chen W., Atyah M., Yin Y., Fu P., Liu S., Hu B., Ren N. (2019). High GCLC Level in Tumor Tissues Is Associated with Poor Prognosis of Hepatocellular Carcinoma after Curative Resection. J. Cancer.

[43] Njålsson R., Norgren S. (2005). Physiological and Pathological Aspects of GSH Metabolism. Acta Paediatr..

[44] Bansal A., Simon M.C. (2018). Glutathione Metabolism in Cancer Progression and Treatment Resistance. J. Cell Biol..

[45] Chen K., Zhang Y., Sedlazeck F.J., Creighton C.J. (2024). Germline Structural Variation Globally Impacts the Cancer Transcriptome Including Disease-Relevant Genes. Cell Rep. Med..

[46] Njålsson R., Carlsson K.S., Winkler A., Larsson A., Norgren S. (2003). Diagnostics in Patients with Glutathione Synthetase Deficiency but without Mutations in the Exons of the GSS Gene. Hum. Mutat..

[47] Li X., Yuan D., Liu Y., Ma Y., Song J., Wang Q., Yang Y. (2015). Five Chinese Patients with 5-Oxoprolinuria Due to Glutathione Synthetase and 5-Oxoprolinase Deficiencies. Brain Dev..

[48] Liu X., Cao Z., Wang W., Zhang C., Wang Y., Pan L., Jia B., Zhang K., Zhang W., Li W. (2023). Engineered Extracellular Vesicle-Delivered CRISPR/CAS9 for Radiotherapy Sensitization of Glioblastoma. ACS Nano.

[49] Ke H., Lin J., Ye Y., Wu W.J., Lin H., Wei H., Huang M., Chang D.W., Dinney C.P., Wu X. (2015). Genetic Variations in Glutathione Pathway Genes Predict Cancer Recurrence in Patients Treated with Transurethral Resection and Bacillus Calmette–Guerin Instillation for Non-Muscle Invasive Bladder Cancer. Ann. Surg. Oncol..

[50] Strohkamp S., Gemoll T., Humborg S., Hartwig S., Lehr S., Freitag-Wolf S., Becker S., Franzén B., Pries R., Wollenberg B. (2017). Protein Levels of Clusterin and Glutathione Synthetase in Platelets Allow for Early Detection of Colorectal Cancer. Cell Mol. Life Sci..

[51] Zhang Y., Zhang Z., Wu M., Zhang R. (2023). Advances and Perspectives of Responsive Probes for Measuring γ-Glutamyl Transpeptidase. ACS Meas. Sci. Au..

[52] Mitrić A., Castellano I. (2023). Targeting Gamma-Glutamyl Transpeptidase: A Pleiotropic Enzyme Involved in Glutathione Metabolism and in the Control of Redox Homeostasis. Free Radic. Biol. Med..

[53] Takemura K., Board P.G., Koga F. (2021). A Systematic Review of Serum γ-Glutamyltransferase as a Prognostic Biomarker in Patients with Genitourinary Cancer. Antioxidants.

[54] Park S., Li Y., Chang L., Tian X. (2019). Prognostic and Clinicopathological Significance of Gamma-Glutamyltransferase in Patients with Hepatocellular Carcinoma. Medicine.

[55] Takemura K., Ito M., Nakanishi Y., Kataoka M., Sakamoto K., Suzuki H., Tobisu K., Koga F. (2019). Serum γ-Glutamyltransferase as a Prognostic Biomarker in Metastatic Castration-Resistant Prostate Cancer Treated with Enzalutamide. Anticancer. Res..

[56] Takemura K., Fukushima H., Ito M., Kataoka M., Nakanishi Y., Sakamoto K., Suzuki H., Tobisu K., Koga F. (2019). Prognostic Significance of Serum γ-Glutamyltransferase in Patients with Advanced Urothelial Carcinoma. Urol. Oncol..

[57] King J.B., West M.B., Cook P.F., Hanigan M.H. (2009). A Novel, Species-Specific Class of Uncompetitive Inhibitors of γ-Glutamyl Transpeptidase. J. Biol. Chem..

[58] Lu E., Wolfreys F., Muppidi J.R., Xu Y., Cyster J.G. (2019). S-Geranylgeranyl-l-Glutathione Is a Ligand for Human B Cell-Confinement Receptor P2RY8. Nature.

[59] Pascale R.M., Simile M.M., Calvisi D.F., Feo C.F., Feo F. (2022). S-Adenosylmethionine: From the Discovery of Its Inhibition of Tumorigenesis to Its Use as a Therapeutic Agent. Cells.

[60] Gao Y., Zhu Y., Awakawa T., Abe I. (2024). Unusual Cysteine Modifications in Natural Product Biosynthesis. RSC Chem. Biol..

[61] Hakimi A.A., Reznik E., Lee C., Creighton C.J., Brannon A.R., Luna A., Aksoy B.A., Liu E.M., Shen R., Lee W. (2016). An Integrated Metabolic Atlas of Clear Cell Renal Cell Carcinoma. Cancer Cell.

[62] Clasen J., Heath A.K., Van Puyvelde H., Huybrechts I., Park J.Y., Ferrari P., Scélo G., Ulvik A., Midttun Ø., Ueland P.M. (2022). Biomarkers of the Transsulfuration Pathway and Risk of Renal Cell Carcinoma in the European Prospective Investigation into Cancer and Nutrition (EPIC) Study. Int. J. Cancer.

[63] Fantone S., Piani F., Olivieri F., Rippo M.R., Sirico A., Di Simone N., Marzioni D., Tossetta G. (2024). Role of SLC7A11/XCT in Ovarian Cancer. Int. J. Mol. Sci..

[64] Jyotsana N., Ta K.T.L., DelGiorno K.E. (2022). The Role of Cystine/Glutamate Antiporter SLC7A11/XCT in the Pathophysiology of Cancer. Front. Oncol..

[65] Koch K., Hartmann R., Suwala A., Rios D.H., Kamp M.A., Sabel M., Steiger H., Willbold D., Schmidt-Wolf I.G.H., Kahlert U.D. (2021). Overexpression of Cystine/Glutamate Antiporter xCT Correlates with Nutrient Flexibility and ZEB1 Expression in Highly Clonogenic Glioblastoma Stem-like Cells (GSCs). Cancers.

[66] Lim J., Delaidelli A., Minaker S.W., Zhang H., Čolović M., Yang H., Negri G.L., Von Karstedt S., Lockwood W.W., Schaffer P. (2019). Cystine/Glutamate Antiporter xCT (SLC7A11) Facilitates Oncogenic RAS Transformation by Preserving Intracellular Redox Balance. Proc. Natl. Acad. Sci. USA.

[67] Mesclon F., Lambert-Langlais S., Carraro V., Parry L., Hainault I., Jousse C., Maurin A., Bruhat A., Fafournoux P., Avérous J. (2017). Decreased ATF4 Expression as a Mechanism of Acquired Resistance to Long-Term Amino Acid Limitation in Cancer Cells. Oncotarget.

[68] Lee J., Roh J. (2022). SLC7A11 as a Gateway of Metabolic Perturbation and Ferroptosis Vulnerability in Cancer. Antioxidants.

[69] Zhang B., Hou Q., Zhang X., Ma Y., Yuan J., Li S., Zhao X., Sun L., Wang H., Zheng H. (2023). Anesthetic Propofol Inhibits Ferroptosis and Aggravates Distant Cancer Metastasis via Nrf2 Upregulation. Free Radic. Biol. Med..

[70] Kilberg M.S., Shan J., Su N. (2009). ATF4-Dependent Transcription Mediates Signaling of Amino Acid Limitation. Trends Endocrinol. Metab..

[71] De La Vega M.R., Chapman E., Zhang D.D. (2018). NRF2 and the Hallmarks of Cancer. Cancer Cell.

[72] Iqbal M.J., Kabeer A., Abbas Z., Siddiqui H.A., Calina D., Sharifi-Rad J., Cho W.C. (2024). Interplay of Oxidative Stress, Cellular Communication and Signaling Pathways in Cancer. Cell Commun. Signal..

[73] Jiang L., Kon N., Li T., Wang S.-J., Su T., Hibshoosh H., Baer R., Gu W. (2015). Ferroptosis as a P53-Mediated Activity during Tumour Suppression. Nature.

[74] Wang L., Liu Y., Du T., Yang H., Lei L., Guo M., Ding H., Zhang J., Wang H., Chen X. (2019). ATF3 Promotes Erastin-Induced Ferroptosis by Suppressing System Xc–. Cell Death Differ..

[75] Bi R., Hu R., Jiang L., Wen B., Jiang Z., Liu H., Mei J. (2023). Butyrate Enhances Erastin-induced Ferroptosis of Lung Cancer Cells via Modulating the ATF3/SLC7A11 pathway. Environ. Toxicol..

[76] Shin C.S., Mishra P., Watrous J.D., Carelli V., D’Aurelio M., Jain M., Chan D.C. (2017). The Glutamate/Cystine xCT Antiporter Antagonizes Glutamine Metabolism and Reduces Nutrient Flexibility. Nat. Commun..

[77] Liu J., Xia X., Huang P. (2020). XCT: A Critical Molecule That Links Cancer Metabolism to Redox Signaling. Mol. Ther..

[78] Bonifácio V.D.B., Pereira S.A., Serpa J., Vicente J.B. (2020). Cysteine Metabolic Circuitries: Druggable Targets in Cancer. Br. J. Cancer.

[79] Zhang J., Pavlova N.N., Thompson C.B. (2017). Cancer Cell Metabolism: The Essential Role of the Nonessential Amino Acid, Glutamine. EMBO J..

[80] Yang L., Venneti S., Nagrath D. (2017). Glutaminolysis: A Hallmark of Cancer Metabolism. Annu. Rev. Biomed. Eng..

[81] Gao P., Tchernyshyov I., Chang T.C., Lee Y.S., Kita K., Ochi T., Zeller K., De Marzo A.M., Van Eyk J.E., Mendell J.T. (2009). C-Myc Suppression of miR-23a/b Enhances Mitochondrial Glutaminase Expression and Glutamine Metabolism. Nature.

[82] Altman B.J., Stine Z.E., Dang C.V. (2016). From Krebs to Clinic: Glutamine Metabolism to Cancer Therapy. Nat. Rev. Cancer.

[83] Locasale J.W., Grassian A., Melman T., Lyssiotis C.A., Mattaini K., Bass A.J., Heffron G.J., Metallo C.M., Muranen T., Sharfi H. (2011). Phosphoglycerate Dehydrogenase Diverts Glycolytic Flux and Contributes to Oncogenesis. Nat. Genet..

[84] Jain M., Nilsson R., Sharma S., Madhusudhan N., Kitami T., Souza A., Kafri R., Kirschner M.W., Clish C.B., Mootha V.K. (2012). Metabolite Profiling Identifies a Key Role for Glycine in Rapid Cancer Cell Proliferation. Science.

[85] Ghanem N., El-Baba C., Araji K., El-Khoury R., Usta J., Darwiche N. (2021). The Pentose Phosphate Pathway in Cancer: Regulation and Therapeutic Opportunities. Chemotherapy.

[86] Li B., Qiu B., Lee D.S.M., Walton Z.E., Ochocki J.D., Mathew L.K., Mancuso A., Gade T.P.F., Keith B., Nissim I. (2014). Fructose-1,6-Bisphosphatase Opposes Renal Carcinoma Progression. Nature.

[87] Yang H., Chen D., Wu Y., Zhou H., Diao W., Liu G., Li Q. (2023). A Feedback Loop of PPP and PI3K/AKT Signal Pathway Drives Regorafenib-Resistance in HCC. Cancer Metab..

[88] Jaganjac M., Milkovic L., Sunjic S.B., Zarkovic N. (2020). The NRF2, Thioredoxin, and Glutathione System in Tumorigenesis and Anticancer Therapies. Antioxidants.

[89] Romero R., Sayin V.I., Davidson S.M., Bauer M.R., Singh S.X., LeBoeuf S.E., Karakousi T.R., Ellis D.C., Bhutkar A., Sánchez-Rivera F.J. (2017). Keap1 Loss Promotes Kras-Driven Lung Cancer and Results in Dependence on Glutaminolysis. Nat. Med..

[90] Wang R., Liang L., Matsumoto M., Iwata K., Umemura A., He F. (2023). Reactive Oxygen Species and NRF2 Signaling, Friends or Foes in Cancer?. Biomolecules.

[91] Vernier M., Dufour C.R., McGuirk S., Scholtes C., Li X., Bourmeau G., Kuasne H., Park M., St-Pierre J., Audet-Walsh E. (2020). Estrogen-Related Receptors Are Targetable ROS Sensors. Genes Dev..

[92] Cuperlovic-Culf M., Cormier K., Touaibia M., Reyjal J., Robichaud S., Belbraouet M., Turcotte S. (2016). 1H NMR Metabolomics Analysis of Renal Cell Carcinoma Cells: Effect of VHL Inactivation on Metabolism. Int. J. Cancer.

[93] Flohé L., Toppo S., Orian L. (2022). The Glutathione Peroxidase Family: Discoveries and Mechanism. Free Radic. Biol. Med..

[94] Brigelius-Flohé R., Maiorino M. (2013). Glutathione Peroxidases. Biochim. Biophys. Acta.

[95] Barbosa N.V., Nogueira C.W., Nogara P.A., De A.F., Aschner M. (2017). Organoselenium Compounds as Mimics of Selenoproteins and Thiol Modifier Agents. Metallomics.

[96] Vašková J., Kočan L., Vaŝko L., Perjési P. (2023). Glutathione-Related Enzymes and Proteins: A Review. Molecules.

[97] Deponte M. (2013). Glutathione Catalysis and the Reaction Mechanisms of Glutathione-Dependent Enzymes. Biochim. Biophys. Acta.

[98] Flohé L., Toppo S., Cozza G., Ursini F. (2011). A Comparison of Thiol Peroxidase Mechanisms. Antioxid. Redox Signal..

[99] Toppo S., Flohé L., Ursini F., Vanin S., Maiorino M. (2009). Catalytic Mechanisms and Specificities of Glutathione Peroxidases: Variations of a Basic Scheme. Biochim. Biophys. Acta.

[100] Zhang W., Liu Y., Yan L., Zhu C., Zou Z. (2024). GPX4, Ferroptosis, and Diseases. Biomed. Pharmacother..

[101] Kipp A.P. (2017). Selenium-dependent glutathione peroxidases during tumor development. Adv. Cancer Res..

[102] Brigelius-Flohé R., Flohé L. (2020). Regulatory Phenomena in the Glutathione Peroxidase Superfamily. Antioxid. Redox Signal..

[103] Miwa T., Adachi T., Ito Y., Hirano K., Sugiura M. (1983). Purification and Properties of Glutathione Peroxidase from Human Liver. Chem. Pharm. Bull..

[104] Handy D.E., Loscalzo J. (2022). The Role of Glutathione Peroxidase-1 in Health and Disease. Free Radic. Biol. Med..

[105] Wei R., Qiu H., Xu J., Mo J., Liu Y., Gui Y., Huang G., Zhang S., Yao H., Huang X. (2020). Expression and Prognostic Potential of GPX1 in Human Cancers Based on Data Mining. Ann. Transl. Med..

[106] Gouazé V., Mirault M.-É., Carpentier S., Salvayre R., Levade T., Andrieu-Abadie N. (2001). Glutathione Peroxidase-1 Overexpression Prevents Ceramide Production and Partially Inhibits Apoptosis in Doxorubicin-Treated Human Breast Carcinoma Cells. Mol. Pharmacol..

[107] Gan X., Chen B., Shen Z., Liu Y., Li H., Xie X., Xu X., Li H., Huang Z., Chen J. (2014). High GPX1 expression promotes esophageal squamous cell carcinoma invasion, migration, proliferation and cisplatin-resistance but can be reduced by vitamin D. Int. J. Clin. Exp. Med..

[108] Huang Z., Liu Y., Huang Z., Li H., Gan X., Shen Z. (2016). 1,25-Dihydroxyvitamin D3 Alleviates Salivary Adenoid Cystic Carcinoma Progression by Suppressing GPX1 Expression through the NF-κB Pathway. Int. J. Oncol..

[109] Liu J., Hinkhouse M.M., Sun W., Weydert C.J., Ritchie J.M., Oberley L.W., Cullen J.J. (2004). Redox Regulation of Pancreatic Cancer Cell Growth: Role of Glutathione Peroxidase in the Suppression of the Malignant Phenotype. Hum. Gene Ther..

[110] Meng Q., Shi S., Liang C., Liang D., Hua J., Zhang B., Xu J., Lei Y. (2018). Abrogation of Glutathione Peroxidase-1 Drives EMT and Chemoresistance in Pancreatic Cancer by Activating ROS-Mediated Akt/GSK3β/Snail Signaling. Oncogene.

[111] Lee E., Choi A., Jun Y., Kim N., Yook J.I., Kim S.Y., Lee S., Kang S.W. (2020). Glutathione Peroxidase-1 Regulates Adhesion and Metastasis of Triple-Negative Breast Cancer Cells via FAK Signaling. Redox Biol..

[112] Lee S.-M., Lee E.K., Kang D.H., Lee J., Hong S.H., Jeong W., Kang S.W. (2021). Glutathione Peroxidase-1 Regulates ASK1-Dependent Apoptosis via Interaction with TRAF2 in RIPK3-Negative Cancer Cells. Exp. Mol. Med..

[113] Cheng Y., Xu T., Li S., Ruan H. (2019). GPX1, a Biomarker for the Diagnosis and Prognosis of Kidney Cancer, Promotes the Progression of Kidney Cancer. Aging.

[114] Yang W., Shen Y., Wei J., Liu F. (2015). MicroRNA-153/Nrf-2/GPx1 Pathway Regulates Radiosensitivity and Stemness of Glioma Stem Cells via Reactive Oxygen Species. Oncotarget.

[115] Chen B., Shen Z., Wu D., Xie X., Xu X., Lv L., Dai H., Chen J., Gan X. (2019). Glutathione Peroxidase 1 Promotes NSCLC Resistance to Cisplatin via ROS-Induced Activation of PI3K/AKT Pathway. BioMed Res. Int..

[116] Min S.Y., Kim H.S., Jung E.J., Jung E.J., Jee C.D., Kim W.H. (2012). Prognostic significance of glutathione peroxidase 1 (GPX1) downregulation and correlation with aberrant promoter methylation in human gastric cancer. Anticancer Res..

[117] Meng Q., Xu J., Liang C., Liu J., Hua J., Zhang Y., Ni Q., Shi S., Lei Y. (2018). GPx1 Is Involved in the Induction of Protective Autophagy in Pancreatic Cancer Cells in Response to Glucose Deprivation. Cell Death Dis..

[118] Esworthy R.S., Doroshow J.H., Chu F. (2022). The Beginning of GPX2 and 30 Years Later. Free Radic. Biol. Med..

[119] Hashinokuchi A., Matsubara T., Ono Y., Shunichi S., Matsudo K., Nagano T., Kinoshita F., Akamine T., Kohno M., Takenaka T. (2024). Clinical and Prognostic Significance of Glutathione Peroxidase 2 in Lung Adenocarcinoma. Ann. Surg. Oncol..

[120] Peng F., Xu Q., Jing X., Chi X., Zhang Z., Meng X., Liu X., Yan J., Li X., Shao S. (2023). GPX2 Promotes EMT and Metastasis in Non-small Cell Lung Cancer by Activating PI3K/AKT/mTOR/Snail Signaling Axis. FASEB Bioadv..

[121] Naiki T., Naiki-Ito A., Iida K., Etani T., Kato H., Suzuki S., Yamashita Y., Kawai N., Yasui T., Takahashi S. (2018). GPX2 Promotes Development of Bladder Cancer with Squamous Cell Differentiation through the Control of Apoptosis. Oncotarget.

[122] Brzozowa-Zasada M., Ianaro A., Piecuch A., Michalski M., Matysiak N., Stęplewska K. (2023). Immunohistochemical Expression of Glutathione Peroxidase-2 (GPX-2) and Its Clinical Relevance in Colon Adenocarcinoma Patients. Int. J. Mol. Sci..

[123] Yang M., Zhu X., Shen Y., He Q., Qin Y., Shao Y., Yuan L., Ye H. (2022). GPX2 Predicts Recurrence-Free Survival and Triggers the Wnt/β-Catenin/EMT Pathway in Prostate Cancer. PeerJ.

[124] Ren Z., Liang H., Galbo P.M., Dharmaratne M., Kulkarni A., Fard A.T., Aoun M.L., Martínez–López N., Suyama K., Benard O. (2022). Redox Signaling by Glutathione Peroxidase 2 Links Vascular Modulation to Metabolic Plasticity of Breast Cancer. Proc. Natl. Acad. Sci. USA.

[125] Brigelius-Flohé R., Kipp A. (2009). Glutathione Peroxidases in Different Stages of Carcinogenesis. Biochim. Biophys. Acta.

[126] Geng D., Zhou Y., Wang M. (2024). Advances in the Role of GPX3 in Ovarian Cancer (Review). Int. J. Oncol..

[127] Wei J., Xie Q., Liu X., Wan C., Wu W., Fang K., Yao Y., Cheng P., Deng D., Liu Z. (2020). Identification the Prognostic Value of Glutathione Peroxidases Expression Levels in Acute Myeloid Leukemia. Ann. Transl. Med..

[128] Liu Q., Bai W., Huang F., Tang J., Lin X. (2019). Downregulation of microRNA-196a Inhibits Stem Cell Self-Renewal Ability and Stemness in Non-Small-Cell Lung Cancer through Upregulating GPX3 Expression. Int. J. Biochem. Cell Biol..

[129] Worley B.L., Kim Y.S., Mardini J., Zaman R., Leon K.E., Vallur P.G., Nduwumwami A.J., Warrick J.I., Timmins P.F., Kesterson J.P. (2019). GPx3 Supports Ovarian Cancer Progression by Manipulating the Extracellular Redox Environment. Redox Biol..

[130] He Q., Chen N., Wang X., Li P., Liu L., Zheng R., Liu W., Jiang K., Zhao J. (2023). Prognostic Value and Immunological Roles of GPX3 in Gastric Cancer. Int. J. Med. Sci..

[131] Yi Z., Jiang L., Zhao L., Zhou M., Ni Y., Yang Y., Yang H., Yang L., Zhang Q., Kuang Y. (2019). Glutathione Peroxidase 3 (GPX3) Suppresses the Growth of Melanoma Cells through Reactive Oxygen Species (ROS)-dependent Stabilization of Hypoxia-inducible Factor 1-α and 2-α. J. Cell Biochem..

[132] Lee S.-H., Golinska M.A., Griffiths J.R. (2021). HIF-1-Independent Mechanisms Regulating Metabolic Adaptation in Hypoxic Cancer Cells. Cells.

[133] Hu Q., Chen J., Yang W.B., Xu M., Zhou J., Tan J., Huang T. (2023). GPX3 Expression Was Down-Regulated but Positively Correlated with Poor Outcome in Human Cancers. Front. Oncol..

[134] Savić Ž., Ćorić V., Vidović S., Vidović V., Bećarević J., Milovač I., Reljic Z., Mirjanić-Azarić B., Škrbić R., Gajanin R. (2023). GPX3 Rs8177412 Polymorphism Modifies Risk of Upper Urothelial Tumors in Patients with Balkan Endemic Nephropathy. Medicina.

[135] Noci S., Dugo M., Bertola F., Melotti F., Vannelli A., Dragani T.A., Galvan A. (2015). A Subset of Genetic Susceptibility Variants for Colorectal Cancer Also Has Prognostic Value. Pharmacogenomics J..

[136] Zhang H., Zhao W., Gu D., Du M., Gong W., Tan Y., Wang M., Wen J., Zhai Y., Xu Z. (2018). Association of Antioxidative Enzymes Polymorphisms with Efficacy of Platin and Fluorouracil-Based Adjuvant Therapy in Gastric Cancer. Cell Physiol. Biochem..

[137] Wang J., Yang I., Wu D., Huang S.-W., Wu J., Juo S.H. (2010). Functional Glutathione Peroxidase 3 Polymorphisms Associated with Increased Risk of Taiwanese Patients with Gastric Cancer. Clin. Chim. Acta.

[138] Zhang Y., Yang Y., Kuang S., Zhang Y., Qin H., Xie J.-F. (2024). GPX3-Mediated Oxidative Stress Affects Pyrimidine Metabolism Levels in Stomach Adenocarcinoma via the AMPK/MTOR Pathway. Int. J. Clin. Pract..

[139] Wang Z., Zhu J., Liu Y., Wang Z., Cao X., Gu Y. (2022). Tumor-Polarized GPX3 + AT2 Lung Epithelial Cells Promote Premetastatic Niche Formation. Proc. Natl. Acad. Sci. USA.

[140] Ma Y., Zhang L., Gao X., Zhu D. (2024). GPX3 Represses Pancreatic Cancer Cell Proliferation, Migration and Invasion, and Improves Their Chemo-sensitivity by Regulating the JNK/c-Jun Signaling Pathway. Exp. Ther. Med..

[141] Cai M., Sikong Y., Wang Q., Zhu S., Pang F., Cui X.-D. (2019). Gpx3 Prevents Migration and Invasion in Gastric Cancer by Targeting NFкB/Wnt5a/JNK Signaling. Int. J. Clin. Exp. Pathol..

[142] Mosca L., Ilari A., Fazi F., Assaraf Y.G., Colotti G. (2021). Taxanes in Cancer Treatment: Activity, Chemoresistance and Its Overcoming. Drug Resist. Updat..

[143] Kelner M.J., Montoya M.A. (1998). Structural Organization of the Human Selenium-Dependent Phospholipid Hydroperoxide Glutathione Peroxidase Gene (GPX4): Chromosomal Localization to 19p13.3. Biochem. Biophys. Res. Commun..

[144] Xie Y., Kang R., Klionsky D.J., Tang D. (2023). GPX4 in Cell Death, Autophagy, and Disease. Autophagy.

[145] Puglisi R., Tramer F., Panfili E., Micali F., Sandri G., Boitani C. (2003). Differential Splicing of the Phospholipid Hydroperoxide Glutathione Peroxidase Gene in Diploid and Haploid Male Germ Cells in the RAT1. Biol. Reprod..

[146] Chen M., Shi Z., Sun Y., Ning H., Xu G., Zhang L. (2023). Prospects for Anti-Tumor Mechanism and Potential Clinical Application Based on Glutathione Peroxidase 4 Mediated Ferroptosis. Int. J. Mol. Sci..

[147] Forcina G.C., Dixon S.J. (2019). GPX4 at the Crossroads of Lipid Homeostasis and Ferroptosis. Proteomics.

[148] Ursini F., Maiorino M. (2020). Lipid Peroxidation and Ferroptosis: The Role of GSH and GPx4. Free Radic. Biol. Med..

[149] Weaver K., Skouta R. (2022). The Selenoprotein Glutathione Peroxidase 4: From Molecular Mechanisms to Novel Therapeutic Opportunities. Biomedicines.

[150] Zhang C., Liu X., Jin S., Chen Y., Guo R. (2022). Ferroptosis in Cancer Therapy: A Novel Approach to Reversing Drug Resistance. Mol. Cancer.

[151] Feng H., Stockwell B.R. (2018). Unsolved Mysteries: How Does Lipid Peroxidation Cause Ferroptosis?. PLoS Biol..

[152] Hassannia B., Vandenabeele P., Vanden Berghe T. (2019). Targeting Ferroptosis to Iron out Cancer. Cancer Cell.

[153] Yang C., Zhang Y., Lin S., Liu Y., Li W. (2021). Correction for: Suppressing the KIF20A/NUAK1/Nrf2/GPX4 Signaling Pathway Induces Ferroptosis and Enhances the Sensitivity of Colorectal Cancer to Oxaliplatin. Aging.

[154] Ni J., Chen K., Zhang J., Zhang X. (2021). Inhibition of GPX4 or mTOR Overcomes Resistance to Lapatinib via Promoting Ferroptosis in NSCLC Cells. Biochem. Biophys. Res. Commun..

[155] Li Y., Li S., Chen Q., Xia T.-L., Luo D.-H., Li L., Liu S.-L., Guo S.-S., Liu L., Du C. (2022). EBV Infection-Induced GPX4 Promotes Chemoresistance and Tumor Progression in Nasopharyngeal Carcinoma. Cell Death Differ..

[156] Battaglia A.M., Chirillo R., Aversa I., Sacco A., Costanzo F., Biamonte F. (2020). Ferroptosis and Cancer: Mitochondria Meet the “Iron Maiden” Cell Death. Cells.

[157] Shen Z., Song J., Yung B.C., Zhou Z., Wu A., Chen X. (2018). Emerging Strategies of Cancer Therapy Based on Ferroptosis. Adv. Mater..

[158] Guo J., Xu B., Han Q., Zhou H., Xia Y., Gong C., Dai X., Li Z., Wu G. (2018). Ferroptosis: A Novel Anti-Tumor Action for Cisplatin. Cancer Res. Treat..

[159] Woo J.H., Shimoni Y., Yang W.S., Subramaniam P.S., Iyer A., Nicoletti P., Martínez M.R., López G., Mattioli M., Realubit R. (2015). Elucidating Compound Mechanism of Action by Network Perturbation Analysis. Cell.

[160] Costa I., Barbosa D.J., Benfeito S., Silva V., Chavarria D., Borges F., Fernando R., Silva R. (2023). Molecular Mechanisms of Ferroptosis and Their Involvement in Brain Diseases. Pharmacol. Ther..

[161] Wang S., Zhang Y., Zhang D., Meng J., Che N., Zhao X., Liu T. (2024). PTGER3 Knockdown Inhibits the Vulnerability of Triple-negative Breast Cancer to Ferroptosis. Cancer Sci..

[162] Yuan M., Chen L., Wang C., Miao Y., Song C., Shi J., Wang L. (2024). AURKA Knockdown Inhibits Esophageal Squamous Cell Carcinoma Progression through Ferroptosis. Heliyon.

[163] Gomaa A.R., Peng D., Chen Z., Soutto M., Abouelezz K.F.M., Corvalán A., El-Rifai W. (2019). Epigenetic Regulation of AURKA by miR-4715-3p in Upper Gastrointestinal Cancers. Sci. Rep..

[164] Cao W., He Y., Lan J., Luo S., Sun B., Xiao C., Yu W., Zeng Z., Lei S. (2024). FOXP3 Promote the Progression of Glioblastoma via Inhibiting Ferroptosis Mediated by Linc00857/miR-1290/GPX4 Axis. Cell Death Dis..

[165] Taylor A.B., Robson A., Houghton B.C., Jepson C.A., Ford W.C.L., Frayne J. (2013). Epididymal Specific, Selenium-Independent GPX5 Protects Cells from Oxidative Stress-Induced Lipid Peroxidation and DNA Mutation. Hum. Reprod..

[166] Tan S., Liu Q., Yang J., Cai J., Yu M., Yu-Bin J. (2022). Macranthoidin B (MB) Promotes Oxidative Stress-Induced Inhibiting of HEPA1-6 Cell Proliferation via Selenoprotein. Biol. Trace Elem. Res..

[167] Rusolo F., Capone F., Pasquale R., Angiolillo A., Colonna G., Castello G., Costantini M., Costantini S. (2017). Comparison of the Seleno-Transcriptome Expression between Human Non-Cancerous Mammary Epithelial Cells and Two Human Breast Cancer Cell Lines. Oncol. Lett..

[168] Mariotti M., Ridge P.G., Zhang Y., Lobanov A., Pringle T.H., Guigó R., Hatfield D.L., Gladyshev V.N. (2012). Composition and Evolution of the Vertebrate and Mammalian Selenoproteomes. PLoS ONE.

[169] Maiorino M., Bosello-Travain V., Cozza G., Miotto G., Roveri A., Toppo S., Zaccarin M., Ursini F. (2015). Understanding Mammalian Glutathione Peroxidase 7 in the Light of Its Homologs. Free Radic. Biol. Med..

[170] Nguyen V.D., Saaranen M.J., Karala A., Lappi A., Wang L., Raykhel I., Alanen H.I., Salo K.E.H., Wang C.-C., Ruddock L.W. (2011). Two Endoplasmic Reticulum PDI Peroxidases Increase the Efficiency of the Use of Peroxide during Disulfide Bond Formation. J. Mol. Biol..

[171] Buday K., Conrad M. (2020). Emerging Roles for Non-Selenium Containing ER-Resident Glutathione Peroxidases in Cell Signaling and Disease. Biol. Chem..

[172] Bosello-Travain V., Conrad M., Cozza G., Negro A., Quartesan S., Rossetto M., Roveri A., Toppo S., Ursini F., Zaccarin M. (2013). Protein Disulfide Isomerase and Glutathione Are Alternative Substrates in the One Cys Catalytic Cycle of Glutathione Peroxidase 7. Biochim. Biophys. Acta.

[173] Wang L., Zhang L., Niu Y., Sitia R., Wang C.C. (2014). Glutathione Peroxidase 7 Utilizes Hydrogen Peroxide Generated by ERO1A to Promote Oxidative Protein Folding. Antioxid. Redox Signal..

[174] Guerriero E., Capone F., Accardo M., Sorice A., Costantini M., Colonna G., Castello G., Costantini S. (2015). GPX4 and GPX7 Over-Expression in Human Hepatocellular Carcinoma Tissues. Eur. J. Histochem..

[175] Chen Z., Hu T., Zhu S., Mukaisho K., El-Rifai W., Peng D.F. (2017). Glutathione Peroxidase 7 Suppresses Cancer Cell Growth and Is Hypermethylated in Gastric Cancer. Oncotarget.

[176] Yao J., Chen X., Liu Z., Zhang R., Zhang C., Yang Q., Yao P., Jiang Q., Wu J., Zhao S. (2021). The Increasing Expression of GPX7 Related to the Malignant Clinical Features Leading to Poor Prognosis of Glioma Patients. Chin. Neurosurg. J..

[177] Zhao Q., Zhang L., Wang Y., Sun Y., Wang T., Cao J., Qi M., Du X., Xia Z., Zhang R. (2022). A Bioinformatic Analysis: The Overexpression and Prognostic Potential of GPX7 in Lower-Grade Glioma. Int. J. Gen. Med..

[178] Ramming T., Hansen H.G., Nagata K., Ellgaard L., Appenzeller-Herzog C. (2014). GPx8 Peroxidase Prevents Leakage of H2O2 from the Endoplasmic Reticulum. Free Radic. Biol. Med..

[179] Yoboue E.D., Rimessi A., Anelli T., Pinton P., Sitia R. (2017). Regulation of Calcium Fluxes by GPX8, a Type-II Transmembrane Peroxidase Enriched at the Mitochondria-Associated Endoplasmic Reticulum Membrane. Antioxid. Redox Signal..

[180] Khatib A., Solaimuthu B., Yosef M.B., Rmaileh A.A., Tanna M., Oren G., Frisch M.S., Axelrod J.H., Lichtenstein M., Shaul Y.D. (2020). The Glutathione Peroxidase 8 (GPX8)/IL-6/STAT3 Axis Is Essential in Maintaining an Aggressive Breast Cancer Phenotype. Proc. Natl. Acad. Sci. USA.

[181] Yang Z., Qin Y., Sun X., Xiong K., Zhu X., Wang Y., Ren Q.-Y., Wu G., Shi-Min W., Cao X. (2022). GPX8 as a Novel Prognostic Factor and Potential Therapeutic Target in Primary Glioma. J. Immunol. Res..

[182] Zhang X., Xu H., Zhang Y., Sun C.-Y., Li Z., Hu C., Zhao D., Guo C. (2022). Immunohistochemistry and Bioinformatics Identify GPX8 as a Potential Prognostic Biomarker and Target in Human Gastric Cancer. Front. Oncol..

[183] Nguyen T.T.M., Nguyen T.H., Kim H.S., Dao T.T.P., Moon Y., Seo M., Kang S.-O., Mai V.-H., An Y., Jung C. (2023). GPX8 Regulates Clear Cell Renal Cell Carcinoma Tumorigenesis through Promoting Lipogenesis by NNMT. J. Exp. Clin. Cancer Res..

[184] Bai Y., Han T., Yan D., Liang C., Gao L., Liu Y., Zhou J., Guo J., Ge D., Wu J. (2024). GPX8+ Cancer-Associated Fibroblast, as a Cancer-Promoting Factor in Lung Adenocarcinoma, Is Related to the Immunosuppressive Microenvironment. BMC Med. Genom..

[185] Hayes J.D., Flanagan J.U., Jowsey I.R. (2005). Glutathione transferases. Annu. Rev. Pharmacol. Toxicol..

[186] Zhang J., Ye Z.-W., Morgenstern R., Townsend D.M., Tew K.D. (2023). Microsomal Glutathione Transferase 1 in Cancer and the Regulation of Ferroptosis. Adv. Cancer Res..

[187] Morel F., Aninat C. (2011). The Glutathione Transferase Kappa Family. Drug Metab. Rev..

[188] Atkinson H.J., Babbitt P.C. (2009). Glutathione Transferases Are Structural and Functional Outliers in the Thioredoxin Fold. Biochemistry.

[189] Board P.G., Coggan M., Chelvanayagam G., Easteal S., Jermiin L.S., Schulte G.K., Danley D.E., Hoth L.R., Griffor M.C., Kamath A.V. (2000). Identification, Characterization, and Crystal Structure of the Omega Class Glutathione Transferases. J. Biol. Chem..

[190] Dourado D., Fernandes P., Ramos M. (2008). Mammalian Cytosolic Glutathione Transferases. Curr. Protein Pept. Sci..

[191] Dourado D.F.a.R., Fernandes P.A., Mannervik B., Ramos M.J. (2008). Glutathione Transferase: New Model for Glutathione Activation. Chemistry.

[192] Singh R.R., Reindl K.M. (2021). Glutathione S-Transferases in Cancer. Antioxidants.

[193] Ketterer B. (1986). Detoxication Reactions of Glutathione and Glutathione Transferases. Xenobiotica.

[194] Singhal S.S., Singh S.P., Singhal P., Horne D., Singhal J., Awasthi S. (2015). Antioxidant Role of Glutathione S-Transferases: 4-Hydroxynonenal, a Key Molecule in Stress-Mediated Signaling. Toxicol. Appl. Pharmacol..

[195] Grussy K., Łaska M., Moczurad W., Król-Kulikowska M., Ściskalska M. (2023). The Importance of Polymorphisms in the Genes Encoding Glutathione S-Transferase Isoenzymes in Development of Selected Cancers and Cardiovascular Diseases. Mol. Biol. Rep..

[196] Liu R.R., Chen J.C., Li M.D., Li T., Tan Y., Zhang M. (2015). A meta-analysis of glutathione S-transferase M1 and T1 genetic polymorphism in relation to susceptibility to nasopharyngeal carcinoma. Int. J. Clin. Exp. Med..

[197] Lalosevic M.S., Coric V., Pekmezovic T., Simic T., Markovic A.P., Ercegovac M.P. (2024). GSTM1 and GSTP1 Polymorphisms Affect Outcome in Colorectal Adenocarcinoma. Medicina.

[198] Lalosevic M.L.j.S., Coric V.M., Pekmezovic T.D., Simic T.P., Ercegovac M.S.P., Markovic A.R.P., Krivokapic Z.V. (2019). Deletion and Single Nucleotide Polymorphisms in Common Glutathione-S Transferases Contribute to Colorectal Cancer Development. Pathol. Oncol. Res..

[199] Elderdery A.Y., Idris H.M.E., Tebien E.M., Diab N.A., Hamza S.M.A., Suliman B.A., Alhamidi A.H., Omer N.E., Mills J. (2023). Impact of GSTT1 and GSTM1 Polymorphisms in the Susceptibility to Philadelphia Negative Chronic Myeloid Leukaemia. Curr. Cancer Drug Targets.

[200] Board P.G., Menon D. (2016). Structure, Function and Disease Relevance of Omega-Class Glutathione Transferases. Arch Toxicol..

[201] Petrovic M., Simic T., Djukic T., Radic T., Savic-Radojevic A., Zekovic M., Durutovic O., Janicic A., Milojevic B., Kajmakovic B. (2023). The Polymorphisms in GSTO Genes (GSTO1 Rs4925, GSTO2 Rs156697, and GSTO2 Rs2297235) Affect the Risk for Testicular Germ Cell Tumor Development: A Pilot Study. Life.

[202] Xu Y.-T., Wang J., Yin R., Qiu M.-T., Xu L., Wang J., Xu L. (2014). Genetic Polymorphisms in Glutathione S-Transferase Omega (GSTO) and Cancer Risk: A Meta-Analysis of 20 Studies. Sci. Rep..

[203] Simic P., Coric V., Pljesa I., Savic-Radojevic A., Zecevic N., Kocic J., Simic T., Pazin V., Pljesa-Ercegovac M. (2024). The Role of Glutathione Transferase Omega-Class Variant Alleles in Individual Susceptibility to Ovarian Cancer. Int. J. Mol. Sci..

[204] Piacentini S., Monaci P.M., Polimanti R., Manfellotto D., Fuciarelli M. (2012). GSTO2*N142D Gene Polymorphism Associated with Hypothyroidism in Italian Patients. Mol. Biol. Rep..

[205] Tiongco R.E., Cayanan N.D., Catacata M., Dominguez M.J. (2024). Ile105Val Polymorphism in the GSTP1 Gene Is Associated with Susceptibility to Acute Myeloid Leukemia: An Updated Systematic Review and Meta-Analysis. Biomarkers.

[206] Feroz Z., Tiwari S., Vijayaraghavalu S., Kumar M. (2023). GSTs Genetic Polymorphism, Gene–Environment Interaction and Association with Gallbladder Cancer Risk in North Indian Population: A Case-Controlled Study. J. Cancer Res. Ther..

[207] Kuang M., Xu W., Cao C.X., Shen L.L., Chang J., Zhang X.L., Chen J.F., Tang C.J. (2016). Glutathione S-Transferase P1 Rs1695 A>G Polymorphism and Breast Cancer Risk: Evidence from a Meta-Analysis. Genet. Mol. Res..

[208] Kim W., Cho Y.-A., Kim D.-C., Lee K.-E. (2022). Association between Genetic Polymorphism of GSTP1 and Toxicities in Patients Receiving Platinum-Based Chemotherapy: A Systematic Review and Meta-Analysis. Pharmaceuticals.

[209] Jiao H., Song A., Cheng L., Zhou D., Luan J., Lin H., Zhang Z. (2024). Association between GSTP1 I105V Polymorphisms and Responses to GSTP1 Inhibitor Treatment: In Silico and in Vitro Insights. J. Biomol. Struct. Dyn..

[210] Kudhair B.K., Alabid N.N., Taheri-Kafrani A., Lafta I.J. (2020). Correlation of GSTP1 Gene Variants of Male Iraqi Waterpipe (Hookah) Tobacco Smokers and the Risk of Lung Cancer. Mol. Biol. Rep..

[211] Wang Z., Qu K., Niu W., Lin T., Xu X., Huang Z., Liu S., Liu S., Chang H., Liu Y. (2015). Glutathione S-Transferase P1 Gene Rs4147581 Polymorphism Predicts Overall Survival of Patients with Hepatocellular Carcinoma: Evidence from an Enlarged Study. Tumor Biol..

[212] Santric V., Djokic M., Suvakov S., Pljesa-Ercegovac M., Nikitovic M., Radic T., Acimovic M., Stankovic V., Bumbasirevic U., Milojevic B. (2020). GSTP1 Rs1138272 Polymorphism Affects Prostate Cancer Risk. Medicina.

[213] Cui J., Li G., Yin J., Li L., Tan Y., Wei H., Liu B., Deng L., Tang J., Chen Y. (2020). GSTP1 and Cancer: Expression, Methylation, Polymorphisms and Signaling (Review). Int. J. Oncol..

[214] Townsend D.M., Tew K.D., He L., King J.B., Hanigan M.H. (2009). Role of Glutathione S-Transferase Pi in Cisplatin-Induced Nephrotoxicity. Biomed. Pharmacother..

[215] Jenderny S., Lin H., Garrett T., Tew K.D., Townsend D.M. (2010). Protective Effects of a Glutathione Disulfide Mimetic (NOV-002) against Cisplatin Induced Kidney Toxicity. Biomed. Pharmacother..

[216] Tew K.D., Monks A., Barone L., Rosser D., Akerman G., Montali J.A., Wheatley J.B., Schmidt D.E. (1996). Glutathione-associated enzymes in the human cell lines of the National Cancer Institute Drug Screening Program. Mol. Pharmacol..

[217] Mousseau M., Chauvin C., Nissou M.F., Chaffanet M., Plantaz D., Pasquier B., Schaerer R., Benabid A. (1993). A study of the expression of four chemoresistance-related genes in human primary and metastatic brain tumours. Eur. J. Cancer.

[218] Wang Z., Liang S., Lian X., Liu L., Zhao S., Xuan Q., Guo L., Liu H., Yang Y., Dong T. (2015). Identification of Proteins Responsible for Adriamycin Resistance in Breast Cancer Cells Using Proteomics Analysis. Sci. Rep..

[219] Dong X., Yang Y., Zhou Y., Bi X., Zhao N., Zhang Z., Li L., Hang Q., Zhang R., Chen D. (2019). Glutathione S-Transferases P1 Protects Breast Cancer Cell from Adriamycin-Induced Cell Death through Promoting Autophagy. Cell Death Differ..

[220] Gurioli G., Martignano F., Salvi S., Costantini M., Gunelli R., Casadio V. (2018). GSTP1 Methylation in Cancer: A Liquid Biopsy Biomarker?. Clin. Chem. Lab. Med..

[221] Ye J., Wu M., He L., Chen P., Liu H., Yang H. (2023). Glutathione-S-Transferase p1 Gene Promoter Methylation in Cell-Free DNA as a Diagnostic and Prognostic Tool for Prostate Cancer: A Systematic Review and Meta-Analysis. Int. J. Endocrinol..

[222] Danos P., Giannoni-Luza S., Carrasco A.G.M., Acosta O., Guevara-Fujita M.L., Concha J.M.C., Miller H.G., Oblitas J.P., Cartagena A.A., Araujo J.M. (2023). Promoter Hypermethylation of RARB and GSTP1 Genes in Plasma Cell-free DNA as Breast Cancer Biomarkers in Peruvian Women. Mol. Genet. Genom. Med..

[223] Wang T., Arifoglu P., Ronai Z., Tew K.D. (2001). Glutathione S-Transferase P1–1 (GSTP1–1) Inhibits c-Jun N-Terminal Kinase (JNK1) Signaling through Interaction with the C Terminus. J. Biol. Chem..

[224] Tew K.D., Townsend D.M. (2012). Glutathione-S-Transferases as Determinants of Cell Survival and Death. Antioxid. Redox Signal..

[225] Wu Y., Fan Y., Xue B., Luo L., Shen J., Zhang S., Jiang Y., Yin Z. (2006). Human Glutathione S-Transferase P1-1 Interacts with TRAF2 and Regulates TRAF2–ASK1 Signals. Oncogene.

[226] Yin S., Zhao S., Li J., Liu K., Ma X., Zhang Z., Wang R., Tian J., Liu F., Song Y. (2023). NUMA1 Modulates Apoptosis of Esophageal Squamous Cell Carcinoma Cells through Regulating ASK1-JNK Signaling Pathway. Cell Mol. Life Sci..

[227] Dorion S., Lambert H., Landry J. (2002). Activation of the P38 Signaling Pathway by Heat Shock Involves the Dissociation of Glutathione S-Transferase Mu from Ask1. J. Biol. Chem..

[228] Romero L., Andrews K., Ng L., O’Rourke K., Maslen A., Kirby G. (2006). Human GSTA1-1 Reduces c-Jun N-Terminal Kinase Signalling and Apoptosis in Caco-2 Cells. Biochem. J..

[229] Liu X., Sui X., Zhang C., Wei K., Bao Y., Xiong J., Zhou Z., Chen Z., Wang C., Zhu H. (2020). Glutathione S-Transferase A1 Suppresses Tumor Progression and Indicates Better Prognosis of Human Primary Hepatocellular Carcinoma. J. Cancer.

[230] Saisawang C., Wongsantichon J., Robinson R.C., Ketterman A.J. (2019). Glutathione Transferase Omega 1-1 (GSTO1-1) Modulates Akt and MEK1/2 Signaling in Human Neuroblastoma Cell SH-SY5Y. Proteins.

[231] Robin S.K.D., Ansari M., Uppugunduri C.R.S. (2020). Spectrophotometric Screening for Potential Inhibitors of Cytosolic Glutathione S-Transferases. J. Vis. Exp..

[232] Allocati N., Masulli M., Di Ilio C., Federici L. (2018). Glutathione Transferases: Substrates, Inihibitors and pro-Drugs in Cancer and Neurodegenerative Diseases. Oncogenesis.

[233] Awasthi S., Srivastava S.K., Ahmad F., Ahmad H., Ansari G.A.S. (1993). Interactions of Glutathione S-Transferase-π with Ethacrynic Acid and Its Glutathione Conjugate. Biochim. Biophys. Acta.

[234] Mulder G.J., Ouwerkerk-Mahadevan S. (1997). Modulation of Glutathione Conjugation in Vivo: How to Decrease Glutathione Conjugation in Vivo or in Intact Cellular Systems in Vitro. Chem. Biol. Interact..

[235] Turella P., Cerella C., Filomeni G., Bullo A., De Maria F., Ghibelli L., Ciriolo M.R., Cianfriglia M., Mattei M., Federici G. (2005). Proapoptotic Activity of New Glutathione S-Transferase Inhibitors. Cancer Res..

[236] Turella P., Filomeni G., Dupuis M.L., Ciriolo M.R., Molinari A., De Maria F., Tombesi M., Cianfriglia M., Federici G., Ricci G. (2006). A Strong Glutathione S-Transferase Inhibitor Overcomes the P-Glycoprotein-Mediated Resistance in Tumor Cells. J. Biol. Chem..

[237] De Luca A., Mei G., Rosato N., Nicolai E., Federici L., Palumbo C., Pastore A., Serra M., Caccuri A.M. (2014). The Fine-Tuning of TRAF2–GSTP1-1 Interaction: Effect of Ligand Binding and in Situ Detection of the Complex. Cell Death Dis..

[238] Sha H., Zou R., Lu Y., Gan Y., Ma R., Feng J., Chen D. (2022). NBDHEX Re-sensitizes Adriamycin-resistant Breast Cancer by Inhibiting Glutathione S-transferase Pi. Cancer Med..

[239] Mahadevan D., Sutton G.R. (2015). Ezatiostat Hydrochloride for the Treatment of Myelodysplastic Syndromes. Expert. Opin. Investig. Drugs.

[240] O’Brien M.L., Vulevic B., Freer S., Boyd J., Shen H., Tew K.D. (1999). Glutathione peptidomimetic drug modulator of multidrug resistance-associated protein. J. Pharmacol. Exp. Ther..

[241] Zhang J., Ye Z.-W., Janssen-Heininger Y., Townsend D.M., Tew K.D. (2021). Development of Telintra as an Inhibitor of Glutathione S-Transferase P. Handb. Exp. Pharmacol..

[242] Lyttle M.H., Satyam A., Hocker M.D., Bauer K.E., Caldwell C.G., Hui H.C., Morgan A.S., Mergia A., Kauvar L.M. (1994). Glutathione-S-Transferase Activates Novel Alkylating Agents. J. Med. Chem..

[243] Dourado D.F.a.R., Fernandes P.A., Ramos M.J., Mannervik B. (2013). Mechanism of Glutathione Transferase P1-1-Catalyzed Activation of the Prodrug Canfosfamide (TLK286, TELCYTA). Biochemistry.

[244] Kavanagh J.J., Gershenson D.M., Choi H., Lewis L., Patel K., Brown G.L., Garcia A., Spriggs D.R. (2005). Multi-Institutional Phase 2 Study of TLK286 (TELCYTA^TM^, a Glutathione S-Transferase P1-1 Activated Glutathione Analog Prodrug) in Patients with Platinum and Paclitaxel Refractory or Resistant Ovarian Cancer. Int. J. Gynecol. Cancer..

[245] Vergote I., Finkler N., Del Campo J., Lohr A., Hunter J., Matei D., Kavanagh J., Vermorken J.B., Meng L., Jones M. (2009). Phase 3 Randomised Study of Canfosfamide (Telcyta®, TLK286) versus Pegylated Liposomal Doxorubicin or Topotecan as Third-Line Therapy in Patients with Platinum-Refractory or -Resistant Ovarian Cancer. Eur. J. Cancer.

[246] Tew K.D. (2005). TLK-286: A Novel glutathioneS-Transferase-Activated Prodrug. Expert. Expert. Opin. Investig. Drugs.

[247] Ismail A., Govindarajan S., Mannervik B. (2024). Human GST P1-1 Redesigned for Enhanced Catalytic Activity with the Anticancer Prodrug Telcyta and Improved Thermostability. Cancers.

[248] Zhang J., Ye Z., Singh S., Townsend D.M., Tew K.D. (2018). An Evolving Understanding of the S-Glutathionylation Cycle in Pathways of Redox Regulation. Free Radic Biol. Med..

[249] Chai Y.C., Mieyal J.J. (2023). Glutathione and Glutaredoxin—Key Players in Cellular Redox Homeostasis and Signaling. Antioxidants.

[250] Oppong D., Schiff W.M., Shivamadhu M.C., Ahn Y. (2023). Chemistry and Biology of Enzymes in Protein Glutathionylation. Curr. Opin. Chem. Biol..

[251] Bechtel T.J., Weerapana E. (2017). From Structure to Redox: The Diverse Functional Roles of Disulfides and Implications in Disease. Proteomics.

[252] Ghezzi P. (2005). Regulation of Protein Function by Glutathionylation. Free Radic. Res..

[253] Janssen-Heininger Y., Nolin J.D., Hoffman S.M., Van Der Velden J., Tully J.E., Lahue K.G., Abdalla S., Chapman D.G., Reynaert N.L., Van Der Vliet A. (2013). Emerging Mechanisms of Glutathione-dependent Chemistry in Biology and Disease. J. Cell. Biochem..

[254] Atkins W.M., Wang R.W., Bird A.W., Newton D.J., Lu A.Y.H. (1993). The Catalytic Mechanism of Glutathione S-Transferase (GST). Spectroscopic Determination of the pKa of Tyr-9 in Rat Alpha 1-1 GST. J. Biol. Chem..

[255] Arnér E.S.J., Holmgren A. (2000). Physiological Functions of Thioredoxin and Thioredoxin Reductase. Eur. J. Biochem..

[256] Findlay V.J., Townsend D.M., Morris T.E., Fraser J.P., He L., Tew K.D. (2006). A Novel Role for Human Sulfiredoxin in the Reversal of Glutathionylation. Cancer Res..

[257] Gallogly M., Mieyal J.J. (2007). Mechanisms of Reversible Protein Glutathionylation in Redox Signaling and Oxidative Stress. Curr. Opin. Pharmacol..

[258] Ogata F.T., Branco V., Vale F.F., Coppo L. (2021). Glutaredoxin: Discovery, Redox Defense and Much More. Redox Biol..

[259] Beer S.M., Taylor E., Brown S., Dahm C.C., Costa N.J., Runswick M.J., Murphy M.P. (2004). Glutaredoxin 2 Catalyzes the Reversible Oxidation and Glutathionylation of Mitochondrial Membrane Thiol Proteins. J. Biol. Chem..

[260] Ukuwela A.A., Bush A.I., Wedd A.G., Xiao Z. (2017). Reduction Potentials of Protein Disulfides and Catalysis of Glutathionylation and Deglutathionylation by Glutaredoxin Enzymes. Biochem. J..

[261] Pal D., Rai A., Checker R., Patwardhan R.S., Singh B., Sharma D., Sandur S.K. (2021). Role of Protein S-Glutathionylation in Cancer Progression and Development of Resistance to Anti-Cancer Drugs. Arch. Biochem. Biophys..

[262] Peltoniemi M., Karala A., Jurvansuu J., Kinnula V.L., Ruddock L.W. (2006). Insights into Deglutathionylation Reactions. J. Biol. Chem..

[263] Brzozowa-Zasada M., Piecuch A., Bajdak-Rusinek K., Gołąbek K., Michalski M., Matysiak N., Czuba Z. (2024). A Prognostic Activity of Glutaredoxin 1 Protein (GRX1) in Colon Cancer. Int. J. Mol. Sci..

[264] Cha M., Kim I.-H. (2009). Preferential Overexpression of Glutaredoxin3 in Human Colon and Lung Carcinoma. Cancer Epidemiol..

[265] Li B., Chen M., Lu M., Jiang X.-X., Pan M., Mao J.-W. (2018). Glutaredoxin 3 Promotes Migration and Invasion via the Notch Signalling Pathway in Oral Squamous Cell Carcinoma. Free Radic. Res..

[266] He F., Wei L., Luo W., Liao Z., Li B., Zhou X., Xiao X., You J., Chen Y., Zheng S. (2016). Glutaredoxin 3 Promotes Nasopharyngeal Carcinoma Growth and Metastasis via EGFR/Akt Pathway and Independent of ROS. Oncotarget.

[267] Park J.W., Mieyal J.J., Rhee S.G., Chock P.B. (2009). Deglutathionylation of 2-Cys Peroxiredoxin Is Specifically Catalyzed by Sulfiredoxin. J. Biol. Chem..

[268] Mishra M., Jiang H., Wu L., Chawsheen H.A., Wei Q. (2015). The Sulfiredoxin–Peroxiredoxin (Srx–Prx) Axis in Cell Signal Transduction and Cancer Development. Cancer Lett..

[269] Menon D., Board P.G. (2013). A Role for Glutathione Transferase Omega 1 (GSTO1-1) in the Glutathionylation Cycle. J. Biol. Chem..

[270] Hughes M., Hooftman A., Angiari S., Tummala P., Zasłona Z., Runtsch M.C., McGettrick A.F., Sutton C.E., Diskin C., Rooke M. (2019). Glutathione Transferase Omega-1 Regulates NLRP3 Inflammasome Activation through NEK7 Deglutathionylation. Cell Rep..

[271] Tew K.D., Manevich Y., Grek C.L., Xiong Y., Uys J.D., Townsend D.M. (2011). The Role of Glutathione S-Transferase P in Signaling Pathways and S-Glutathionylation in Cancer. Free Radic. Biol. Med..

[272] Webb B.A., Chimenti M.S., Jacobson M.P., Barber D.L. (2011). Dysregulated pH: A Perfect Storm for Cancer Progression. Nat. Rev. Cancer.

[273] Mailloux R.J. (2020). Protein S-Glutathionylation Reactions as a Global Inhibitor of Cell Metabolism for the Desensitization of Hydrogen Peroxide Signals. Redox Biol..

[274] Van Der Velden J., Kinsey M., Chia S.B., Lahue K.G., Qian X., Janssen-Heininger Y. (2017). GSTP1-Catalyzed PKM2 S-Glutathionylation Regulates Glycolysis in Non-Small Cell Lung Cancer and Is Attenuated with a Clinically Relevant Inhibitor of Glutathione-S-Transferase P. Free Radic Biol. Med..

[275] Adachi T., Pimentel D.R., Heibeck T.H., Hou X., Lee Y.J., Jiang B., Ido Y., Cohen R.A. (2004). S-Glutathiolation of RAS Mediates Redox-Sensitive Signaling by Angiotensin II in Vascular Smooth Muscle Cells. J. Biol. Chem..

[276] Yang Y., Dong X., Zheng S., Sun J., Ye J., Chen J., Fang Y., Zhao B., Yin Z., Cao P. (2020). GSTpi Regulates VE-Cadherin Stabilization through Promoting S-Glutathionylation of Src. Redox Biol..

[277] Abdelsaid M., El-Remessy A.B. (2012). S-Glutathionylation of LMW-PTP Regulates VEGF-Mediated FAK Activation and Endothelial Cell Migration. J. Cell Sci..

[278] Sakai J., Li J., Subramanian K.K., Mondal S., Bajrami B., Hattori H., Jia Y., Dickinson B.C., Zhong J., Ye K. (2012). Reactive Oxygen Species-Induced Actin Glutathionylation Controls Actin Dynamics in Neutrophils. Immunity.

[279] Velu C.S., Niture S.K., Doneanu C.E., Pattabiraman N., Srivenugopal K.S. (2007). Human P53 Is Inhibited by Glutathionylation of Cysteines Present in the Proximal DNA-Binding Domain during Oxidative Stress. Biochemistry.

[280] Seo M., Lee Y.H. (2014). PFKFB3 Regulates Oxidative Stress Homeostasis via Its S-Glutathionylation in Cancer. J. Mol. Biol..

[281] Huang Z., Pinto J.T., Deng H., Richie J.P. (2008). Inhibition of Caspase-3 Activity and Activation by Protein Glutathionylation. Biochem. Pharmacol..

[282] Humphries K.M., Juliano C.E., Taylor S.S. (2002). Regulation of CAMP-Dependent Protein Kinase Activity by Glutathionylation. J. Biol. Chem..

[283] Kawano T., Inokuchi J., Eto M., Murata M., Kang J.H. (2022). Protein Kinase C (PKC) Isozymes as Diagnostic and Prognostic Biomarkers and Therapeutic Targets for Cancer. Cancers.

[284] Benavides F., Blando J., Pérez C.F., Garg R., Conti C.J., DiGiovanni J., Kazanietz M.G. (2011). Transgenic Overexpression of PKCε in the Mouse Prostate Induces Preneoplastic Lesions. Cell Cycle.

[285] Wang H., Gutiérrez-Uzquiza Á., Garg R., Barrio-Real L., Abera M.B., López-Haber C., Rosemblit C., Lu H., Abba M.C., Kazanietz M.G. (2014). Transcriptional Regulation of Oncogenic Protein Kinase CΕ (PKCΕ) by STAT1 and SP1 Proteins. J. Biol. Chem..

[286] Ward N.E., Stewart J.R., Ioannides C.G., O’Brian C.A. (2000). Oxidant-Induced S-Glutathiolation Inactivates Protein Kinase C-A (PKC-A): A Potential Mechanism of PKC Isozyme Regulation. Biochemistry.

[287] Rao R., Clayton L. (2002). Regulation of Protein Phosphatase 2A by Hydrogen Peroxide and Glutathionylation. Biochem. Biophys. Res. Commun..

[288] Liu T., Wang X., Zhang L. (2011). [The Correlation between the up-Regulation of Hsp90 and Drug Resistance to Cisplatin in Lung Cancer Cell Line]. Zhongguo Fei Ai Za Zhi.

[289] Zhou J., Tang J., Sun W., Wang H. (2019). PGK1 Facilities Cisplatin Chemoresistance by Triggering HSP90/ERK Pathway Mediated DNA Repair and Methylation in Endometrial Endometrioid Adenocarcinoma. Mol. Med..

[290] Shih Y.-Y., Lin H., Jan H., Chen Y., Ong L.-L., Yu A.L., Lin C. (2022). S-Glutathionylation of Hsp90 Enhances Its Degradation and Correlates with Favorable Prognosis of Breast Cancer. Redox Biol..

[291] Zhang J., Ye Z., Chen W., Culpepper J., Jiang H., Ball L.E., Mehrotra S., Blumental-Perry A., Tew K.D., Townsend D.M. (2020). Altered Redox Regulation and S-Glutathionylation of BiP Contribute to Bortezomib Resistance in Multiple Myeloma. Free Radic. Biol. Med..

[292] Pfefferle A., Mailloux R.J., Adjeitey C.N.K., Harper M. (2013). Glutathionylation of UCP2 Sensitizes Drug Resistant Leukemia Cells to Chemotherapeutics. Biochim. Biophys. Acta..

[293] Zhang L., Ludden C., Cullen A.J., Tew K.D., De Barros A.L.B., Townsend D.M. (2023). Nuclear Factor Kappa B Expression in Non-Small Cell Lung Cancer. Biomed. Pharmacother..

[294] Butturini E., De Prati A.C., Boriero D., Mariotto S. (2019). Natural Sesquiterpene Lactones Enhance Chemosensitivity of Tumor Cells through Redox Regulation of STAT3 Signaling. Oxid. Med. Cell Longev..

[295] Butturini E., De Prati A.C., Chiavegato G., Rigo A., Cavalieri E., Darra E., Mariotto S. (2013). Mild Oxidative Stress Induces S-Glutathionylation of STAT3 and Enhances Chemosensitivity of Tumoural Cells to Chemotherapeutic Drugs. Free Radic. Biol. Med..

[296] Robertson H., Dinkova-Kostova A.T., Hayes J.D. (2020). NRF2 and the Ambiguous Consequences of Its Activation during Initiation and the Subsequent Stages of Tumourigenesis. Cancers.

[297] Hecht F., Zocchi M., Alimohammadi F., Harris I.S. (2024). Regulation of Antioxidants in Cancer. Mol. Cell..

[298] Holland R.J., Hawkins A., Eggler A.L., Mesecar A.D., Fabris D., Fishbein J.C. (2008). Prospective Type 1 and Type 2 Disulfides of KEAP1 Protein. Chem. Res. Toxicol..

[299] Zhang L., Tew K.D. (2021). Reductive Stress in Cancer. Adv. Cancer Res..

[300] Krakowiak A., Pietrasik S. (2023). New Insights into Oxidative and Reductive Stress Responses and Their Relation to the Anticancer Activity of Selenium-Containing Compounds as Hydrogen Selenide Donors. Biology.

[301] Xiao W., Loscalzo J. (2020). Metabolic Responses to Reductive Stress. Antioxid. Redox Signal..

[302] Kôrge P., Calmettes G., Weiss J.N. (2015). Increased Reactive Oxygen Species Production during Reductive Stress: The Roles of Mitochondrial Glutathione and Thioredoxin Reductases. Biochim. Biophys. Acta Bioenerg..

[303] Chun K., Kim D.-H., Surh Y. (2021). Role of Reductive versus Oxidative Stress in Tumor Progression and Anticancer Drug Resistance. Cells.

